# Fc-Engineering Improves PET Imaging of Anti-Mesothelin VH-Fc across Multiple Tumor Mouse Models and Reveals Sex-Specific Renal Clearance

**DOI:** 10.1021/acs.bioconjchem.5c00591

**Published:** 2025-12-24

**Authors:** Abhinav Bhise, Xiaojie Chu, Anders Josefsson, Angel G. Cortez, George Diehl, Lora H. Rigatti, Hyun Jung Park, Jessie R. Nedrow, Wei Li

**Affiliations:** † Department of Radiology, 12317 University of Pittsburgh School of Medicine, Pittsburgh, Pennsylvania 15213, United States; ‡ 72058 Hillman Cancer Center, University of Pittsburgh School of Medicine, Pittsburgh, Pennsylvania 15213, United States; § Center for Antibody Therapeutics, Division of Infectious Diseases, Department of Medicine, University of Pittsburgh, School of Medicine, Pittsburgh, Pennsylvania 15261, United States; ∥ Division of Laboratory Animal Resources, University of Pittsburgh School of Medicine, Pittsburgh, Pennsylvania 15213, United States; ⊥ Department of Human Genetics, 6614 University of Pittsburgh, Pittsburgh, Pennsylvania 15260, United States

## Abstract

Mesothelin (MSLN) is overexpressed in various malignancies, making it a promising target for molecular imaging and therapeutic strategies. Anti-MSLN VH-Fc fusion proteins show high tumor uptake as compared with monoclonal antibodies; however, elevated accumulation in Fc-rich organs (liver, spleen) can compromise tumor-to-background ratios and limit clinical applicability. To overcome this, we developed Fc mutant anti-MSLN VH-Fc fusion proteins incorporating G236R/L328R (GRLR) and L234A/L235A/P329G (LALAPG) mutations to eliminate FcγRs interactions. Engineered mutants exhibited high purity (>95%), retained strong MSLN binding (KD 2.2–3.7 nM), and effectively silenced FcγR binding by *ex vivo* and *in vivo* analyses. Following zirconium-89 radiolabeling, PET imaging was conducted across multiple xenograft models with varying MSLN expression. In HCT116 xenografts, [^89^Zr]­Zr-2A10-VH-Fc_LALAPG_ demonstrated substantially higher uptake (13.0 ± 0.1%ID/g at 120 h p.i.) than [^89^Zr]­Zr-2A10-VH-Fc_WT_ (4.2 ± 0.6%ID/g), while substantially reducing liver (LALAPG: 4.3 ± 0.6%ID/g vs WT: 19.8 ± 2.8%ID/g) and spleen (LALAPG: 9.3 ± 0.1%ID/g vs WT: 95.0 ± 39.3%ID/g) uptake. Biodistribution studies in additional xenograft models confirmed a high specific uptake for [^89^Zr]­Zr-2A10-VH-Fc_LALAPG_ in tumors with moderate to high MSLN expression. Notably for the mutants, females exhibited higher renal retention than males, indicating sex-dependent pharmacokinetics. These findings highlight Fc-engineered VH-Fc fusion proteins, particularly the LALAPG, as promising agents with enhanced tumor specificity, improved pharmacokinetics, and significantly reduced off-target uptake, supporting their use in PET imaging-guided therapeutic applications.

## Graphical abstract

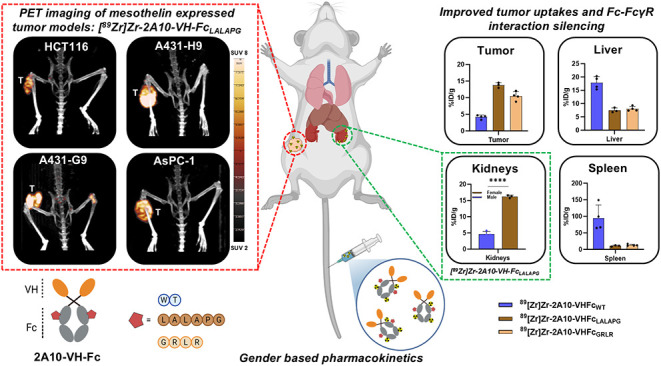

## Introduction

Mesothelin (MSLN) has emerged as a promising cell surface biomarker for targeted imaging and therapies due to its overexpression in different malignancies.^1^ MSLN is glycosylphosphatidylinositol (GPI) anchored protein with low expression in normal tissues and markedly upregulated in several solid tumors, such as mesothelioma, colorectal cancer, pancreatic cancer, lung cancer, and ovarian cancer.^2,3^ Elevated MSLN levels are often correlated with reduced overall survival rates due to poor prognosis, especially when the MSLN expression is heterogeneous throughout the tumor.^4,5^ This variable expression makes MSLN an attractive biomarker for targeted imaging and therapy.

While MSLN is an attractive target for imaging and therapy, conventional monoclonal antibodies face limitations against solid tumors. The large molecular mass increases the hydrodynamic radius and lowers the diffusion coefficient, restricting penetration and distribution within the tumor parenchyma and stroma.^6–8^ These physicochemical limitations necessitate the development of alternative antibody scaffolds to achieve deeper tumor penetration and improved pharmacokinetics (PK) for therapeutic outcomes.

It has been shown that reducing the antibody’s size exponentially increases diffusion through normal and tumor tissues; a 2-fold decrease can yield a 4-fold increase in tissue penetration.^9,10^ Previously, we developed a VH-Fc fusion protein, 2A10-VH-Fc, approximately half the size of a normal IgG.^11^ After radiolabeling with zirconium-89 (^89^Zr), its efficacy was assessed in human colorectal tumor models by positron emission tomography (PET) imaging against m912, an anti-MSLN IgG1. The resulting radioimmunoconjugate, [^89^Zr]­Zr-2A10-VH-Fc fusion protein, exhibited increased tumor uptake and penetration with a better PK profile compared with that of IgG-m912. Despite these improvements, we noticed significant distribution of 2A10-VH-Fc in normal organs like spleen and liver. We hypothesize that this off-target distribution may be due to VH-Fc’s interacting with Fc receptors expressed by tissue-resident immune cells in the reticuloendothelial system, including liver, spleen, and bone marrow.^12–15^ Previous studies have demonstrated that Fc-FcγR interactions significantly influence the *in vivo* behavior of antibody-based radiotracers and immunoconjugates. Zeglis et al. have shown how FcγR engagement can alter antibody biodistribution and imaging performance, emphasizing the importance of understanding Fc-mediated mechanisms in radiopharmaceutical development.^16–18^

FcγR silencing, referred to as engineering of the antibody’s Fc region with mutations that abrogate FcγR binding, has applications in antibody-based therapeutics when the Fc effector function is not needed or undesired, such as tissue damage introduced by FcγR-mediated immune effector functions.^19,20^ These mutations should efficiently eliminate FcγR engagement while preserving WT-like properties like aggregation resistance, thermostability, and immunogenicity.^21^ The G236R/L328R (GRLR) and L234A/L235A/P329G (LALAPG) mutations are leading candidates that have progressed into clinical trials.^22–24^ The bispecific T cell engager glofitamab (anti-CD20 × anti-CD3 IgG1), containing the LALAPG mutation, is approved for relapsed or refractory diffuse large B-cell lymphoma.^25^ The lower hinge G236R mutation disrupts binding to Fcγ receptors (FcγRs; two histidines in FcγRIII in Figure 1A). The G236R mutation combined with the L328R mutation completely abrogates binding to all FcγRs and C1q.^26^ The L234A/L235A mutations break the van der Waals interaction with the FcγRs residues (Threonine 116 and Valine 158 in FcγRIII in Figure 1A), leading to significantly reduced binding to FcγRs. By combining with the P329G mutation, which destroys the side chain hydrophobic packing of Fc P329 with the W90 and W113 of FcγRs (so-called proline sandwich, Figure 1A),^19^ the LALAPG mutation almost abrogates all interactions with FcγRs.^23^

**Figure 1:**
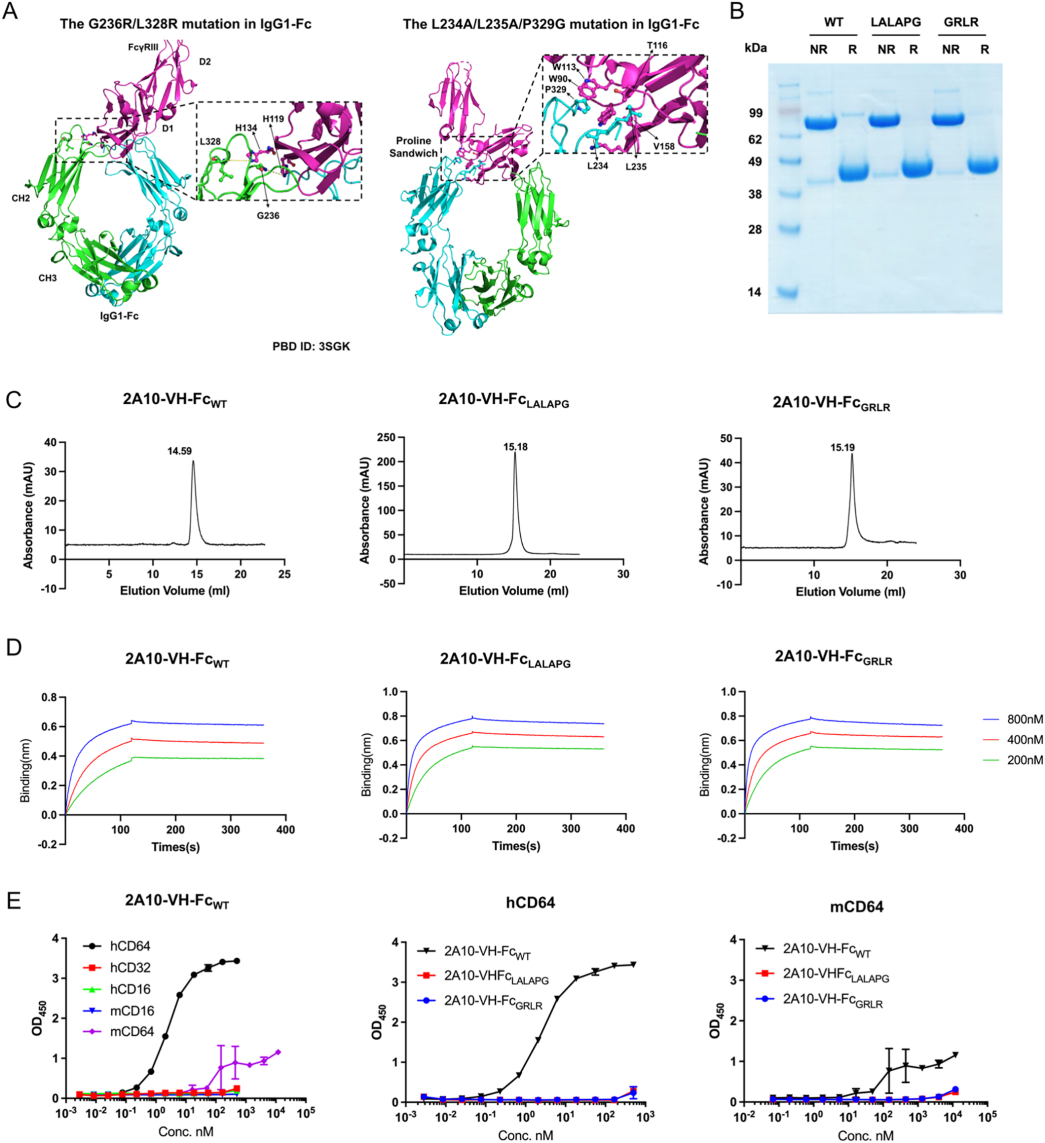
(A) Structural mapping of IgG1-Fc showing GRLR (G236R/L328R) and LALAPG (L234A/L235A/P329G) mutations used to silence FcγR binding (PDB: 3SGK). (B) SDS-PAGE of 2A10-VH-Fc mutants (NR: nonreducing; R: reducing). (C) Size-exclusion chromatography of 2A10-VH-Fc WT, GRLR, and LALAPG mutants. (D) Blitz analysis of 2A10-VH-Fc mutants. (E) Binding affinity of VH-Fc 2A10- (WT, GRLR, and LALAPG) with human and mouse FcγRs.

In this study, we engineered the 2A10-VH-Fc fusion protein with FcγR silencing mutations (GRLR and LALAPG) to explore the impact of Fc-FcR interactions on biodistribution and PK. The resulting variants are designated as 2A10-VH-Fc_WT_, 2A10-VH-Fc_GRLR_, and 2A10-VH-Fc_LALAPG_, hereafter. These VH-Fcs serve as anti-MSLN PET imaging agents, allowing a comparison of their *in vivo* performance in murine models of MSLN-positive solid tumors.

## Results

### Generation and Evaluation of 2A10-VH-Fc Mutants

To reduce nonspecific organ distribution, we engineered Fc mutant VH-Fc 2A10 proteins by introducing site-specific Fc mutations (GRLR and LALAPG). The resulting variants, including the wild-type (2A10-VH-Fc_WT_), 2A10-VH-Fc_GRLR_, and 2A10-VH-Fc_LALAPG_, were purified by size-exclusion chromatography (SEC) and confirmed by SDS-PAGE (Figure 1B), exhibiting ≥95% purity. As observed previously with 2A10-VH-Fc_WT_, both mutants exhibit monomeric folding without high molecular weight species by SEC, demonstrating low aggregation (Figure 1C). Furthermore, equilibrium dissociation constants (KD) of 2A10-VH-Fc_WT_, 2A10-VH-Fc_GRLR_, and 2A10-VH-Fc_LALAPG_ (3.7, 2.2, and 2.5 nM, respectively) confirmed high MSLN affinity, indicating that Fc modifications did not alter the binding avidity (Figure 1D). The liquid chromatography–mass spectrometry (LC/MS) mass analysis demonstrated similar biantennary glycosylation of 2A10-VH-Fc_WT_ and VH-Fc_LALAPG_, with predominant G0F and G1F species (Figure S1), consistent with reports showing LALAPG does not impact IgG1-Fc N297 glycosylation.^19,23^

FcγRs interactions of 2A10-VH-Fc_WT_ and Fc mutants were assessed by ELISA (Figure 1E). The 2A10-VH-Fc_WT_ exhibited affinity toward human and murine CD64 (FcγRI), with minimal to no binding to human CD32 (FcγRII), CD16 (FcγRIII), and mouse CD16. Both mutants exhibited no human CD64 binding, confirming successful silencing of the Fc-FcγRI interaction.

### Bioconjugation, Radiolabeling, and Serum Stabilities of 2A10-VH-Fc Mutants

The 2A10-VH-Fc fusion proteins (WT, GRLR, and LALAPG) were modified with DFO-Bn-NCS at a molar ratio of 1:5 and radiolabeled with zirconium-89 (Figure 2A). All radiolabeled VH-Fc’s were purified, buffer-exchanged with phosphate-buffered saline (PBS), and achieved ≥99% radiopurity by radio-HPLC (Figure 2B). The radiolabeling efficiencies were >95% with molar activities of 1.1–1.5 MBq/μmol (Table S1). *In vitro* stability of [^89^Zr]­Zr-2A10-VH-Fc_LALAPG_ and [^89^Zr]­Zr-2A10-VH-Fc_GRLR_ was evaluated in PBS and human serum (HS) at 37 °C over 3 days (Figure S2 and Table S2). Both mutants showed ∼90% radiochemical stability, comparable to 2A10-VH-Fc_WT_.^11^ Briefly, [^89^Zr]­Zr-2A10-VH-Fc_LALAPG_ displayed 91.1 ± 1.3% (PBS) and 91.2 ± 2.71% (HS) at 24 h, decreasing slightly to 88.9 ± 1.2% (PBS) and 92.5 ± 3.7% (HS) on day 3. Similarly, [^89^Zr]­Zr-2A10-VH-Fc_GRLR_ demonstrated high stability of 91.2 ± 2.7% (PBS) and 91.5 ± 3.5% (HS) at 24 h and 92.9 ± 3.6% (PBS) and 90.2 ± 3.3% (HS) on day 3. These results confirm that the 2A10-VH-Fc mutants retain high stability under physiological and buffer conditions.

**Figure 2:**
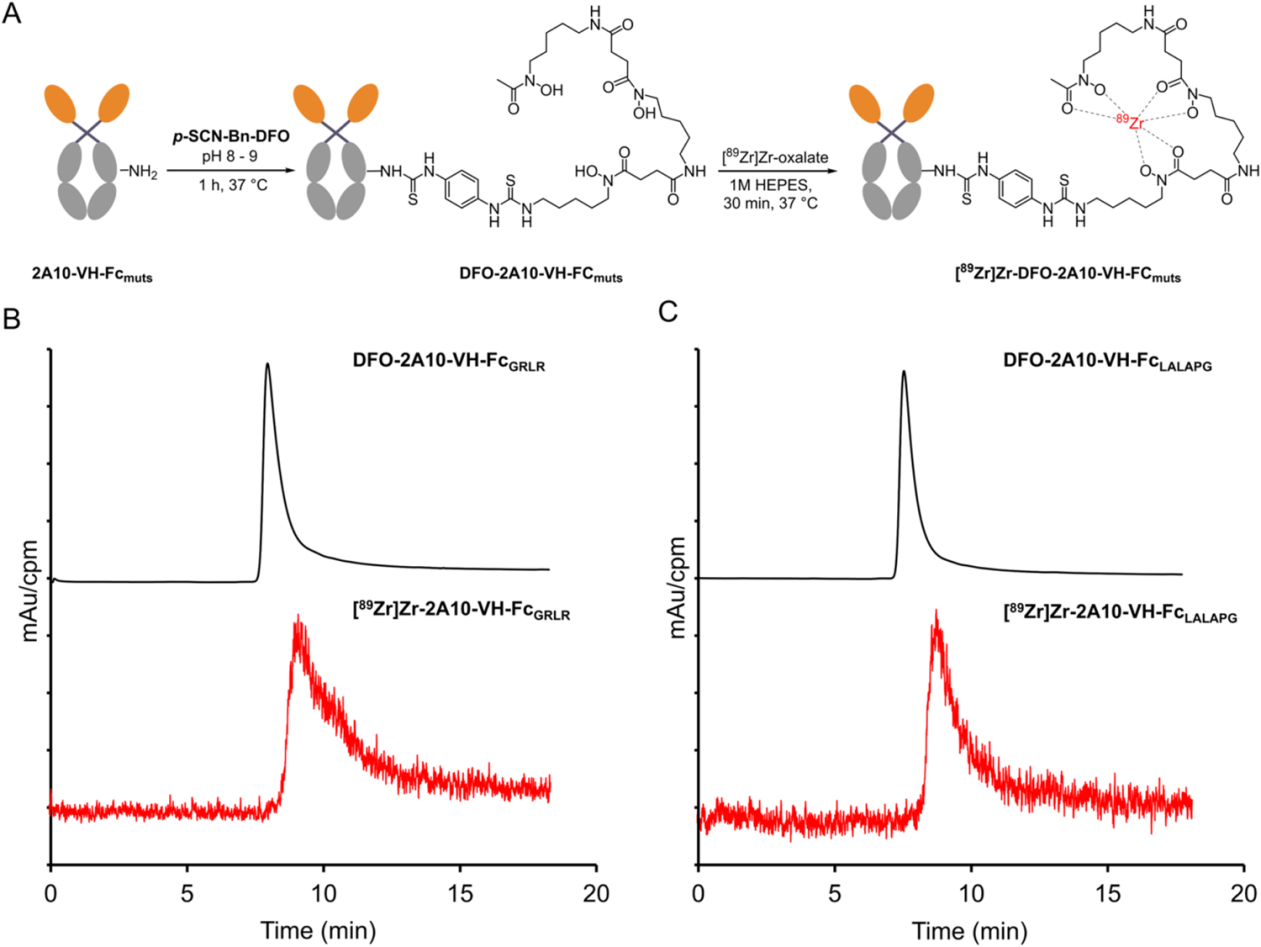
(A) Bioconjugation and radiolabeling of VH-Fc proteins. Overlaid HPLC chromatograms of VH-Fc mutants (B) GRLR and (C) LALAPG with DFO conjugates shown in black and radiolabeled proteins in red. The UV was recorded at 280 nm, while the radio chromatogram is displayed in counts per minute (CPM).

### Preliminary Evaluation of 2A10-VH-Fc Mutants in a Murine Model of Colorectal Cancer

PET imaging and *ex vivo* biodistribution experiments were conducted to compare the PK profiles of [^89^Zr]­Zr-2A10-VH-Fc_WT_, [^89^Zr]­Zr-2A10-VH-Fc_LALAPG_, and [^89^Zr]­Zr-2A10-VH-Fc_GRLR_ in HCT116 tumor-bearing NCG female mice (18–19 weeks) at 90 min, 18/24 h, 48 h, and 120 h (Figure 3). Both [^89^Zr]­Zr-2A10-VH-Fc mutants (LALAPG and GRLR) demonstrated higher tumor accumulation at 120 h postinjection (p.i.) (Figure 3A,B,E). [^89^Zr]­Zr-2A10-VH-Fc_LALAPG_ achieved the highest tumor accumulation (SUV_mean_ 1.69 ± 0.63; 3.78 ± 0.78%ID/g), followed by [^89^Zr]­Zr-2A10-VH-Fc_GRLR_ (1.39 ± 0.29 SUV_mean_; 10.46 ± 1.34%ID/g), while [^89^Zr]­Zr-2A10-VH-Fc_WT_ demonstrated peaked at 48 h p.i. (1.37 ± 0.68 SUV_mean_) but declined at 120 h (0.75 ± 0.20 SUV_mean_; 4.17 ± 0.60%ID/g). Correspondingly, Fc-rich spleen demonstrated lower uptake at 120 h p.i. for both mutants [^89^Zr]­Zr-2A10-VH-Fc_LALAPG_ (10.76 ± 2.12%ID/g) and [^89^Zr]­Zr-2A10-VH-Fc_GRLR_ (12.98 ± 2.62%ID/g), which were significantly lower (*p* < 0.0001) than WT (94.95 ± 39.34%ID/g), confirming effective silencing of Fc-FcγR interaction. Similarly, the liver with the presence of CD64+ macrophages^27^ demonstrated lower uptake at day 5 for the mutants (1.87 ± 0.22 SUV_mean_; 7.50 ± 0.89%ID/g for LALAPG) (1.77 ± 0.16 SUV_mean_; 8.05 ± 0.91%ID/g for GRLR) compared with the WT (4.18 ± 0.46 SUV_mean_; 17.83 ± 2.37%ID/g) (Figures S3 and S4; Tables S3 and S4). At 120 h p.i., kidney uptake was significantly higher for the 2A10-VH-Fc mutants, with [^89^Zr]­Zr-2A10-VH-Fc_LALAPG_ having an SUV_mean_ of 4.71 ± 0.71 and 16.17 ± 0.46%ID/g; *p* < 0.0001, while the [^89^Zr]­Zr-2A10-VH-Fc_GRLR_ exhibited the highest renal retention (7.05 ± 1.23 SUV_mean_; 26.62 ± 4.65%ID/g; *p* < 0.0001) relative to [^89^Zr]­Zr-2A10-VH-Fc_WT_ (1.15 ± 0.18 SUV_mean_; 3.59 ± 0.54%ID/g). Collectively, PET imaging and biodistribution studies demonstrated that 2A10-VH-Fc mutants had enhanced tumor accumulation with reduced liver and spleen uptake. However, the mutants had an elevated renal uptake, prompting further investigation into tracer stability.

**Figure 3:**
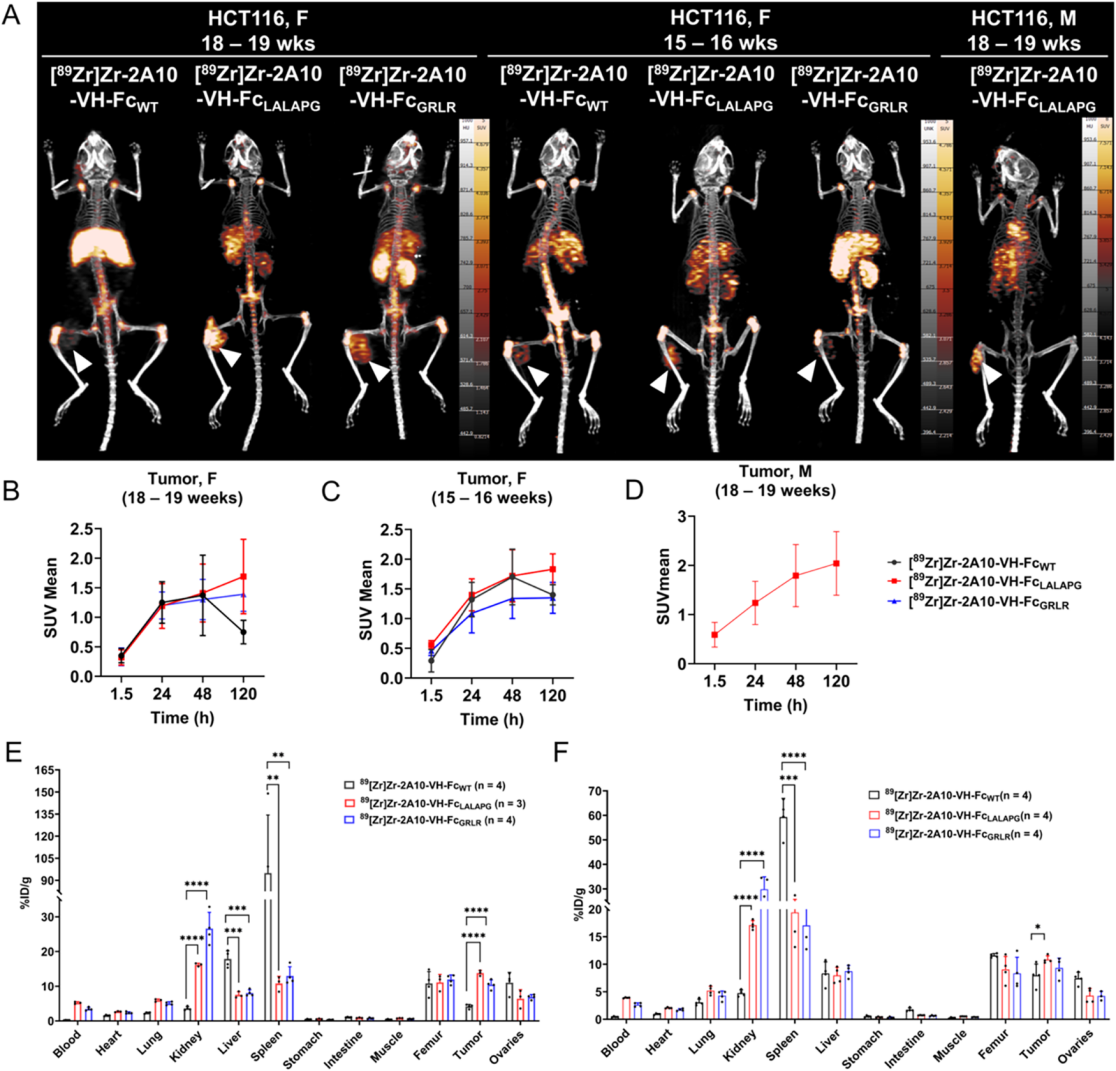
*In vivo* PET/CT imaging and tumor uptake kinetics of [^89^Zr]­Zr-labeled 2A10-VH-Fc fusion proteins in HCT116 xenograft-bearing mice. (A) Representative MIP PET/CT images acquired at 120 h postinjection (p.i.) in female mice (18–19 weeks and 15–16 weeks old) injected with VH-Fc_WT,_ VH-Fc_LALAPG_, or VH-Fc_GRLR_ and male mice (18–19 weeks old) receiving only VH-Fc_LALAPG_. White arrowheads indicate tumor. (B–D) Tumor uptake expressed as SUV_mean_ over time for female (18–19 weeks) (B), female (15–16 weeks) (C), and male (18–19 weeks) (D). Data are shown as mean ± SD (*n* = 3/4 per group) for each construct. Biodistribution of [^89^Zr]­Zr-labeled VH-Fc fusion proteins at 120 h p.i. in HCT116 xenografts (E) female 18–19 weeks and (F) female 15–16 weeks. * *p* ≤ 0.05, ** *p* ≤ 0.01, *** *p* ≤ 0.001, **** *p* ≤ 0.0001.

### Investigating Stabilities and Kidney Deposition of 2A10-VH-Fc Mutants

To assess biochemical integrity of the mutants, *in vivo* stability in blood, kidney, and urine was evaluated in NCG healthy nontumor-bearing mice (*n* = 3). The [^89^Zr]­Zr-2A10-VH-Fc_GRLR_ showed 81.2 ± 5.4% stability in the kidney and 82.7 ± 6.0% in the blood at 90 min p.i., whereas [^89^Zr]­Zr-2A10-VH-Fc_LALAPG_ exhibited higher stability, 92.8 ± 6.3% in the kidney and 96.0 ± 1.3% in the blood at 24 h p.i. (Figure S5 and Table S5). To investigate metabolic degradation contributing to kidney deposition, urine was collected and analyzed at 90 min p.i. by SEC-HPLC (Figure S6). Intact 2A10-VH-Fc (MW = ∼80 kDa) and 2A10-VH-domain (MW = 15 kDa) eluted at ∼10.5 and ∼13.0 min, respectively. Both mutants showed additional prominent peaks at ∼13.5–14 min, that did not correspond to known VH-Fc or VH fragments, indicating formation of metabolites with molecular weights <15 kDa.

#### Strain-Based Kidney Uptake of Fc-Silenced Mutants

To determine whether this retention pattern was specific to NCG mice, kidney uptakes were also evaluated in CD1-IGS mice (males and female; 7–9 weeks) (Figure S7 and Table S6). Across all 2A10-VH-Fc variants, females showed higher renal uptake; however, only Fc mutants showed significantly higher kidney retention. LALAPG females showed 42.78 ± 4.60%ID/g vs 8.73 ± 1.96%ID/g in males (*p* < 0.0001), and GRLR females showed 45.56 ± 14.52%ID/g vs 9.37 ± 3.86%ID/g in males (*p <* 0.0029), consistent with findings in NCG mice. These results confirm that the renal retention pattern is comparable across mouse strains but further indicate sex-based differences.

#### Age-Dependent Biodistribution and Renal Uptake of 2A10-VH-Fc Mutants

To assess age variables, renal uptake was compared between 18–19 weeks (older) and 15–16 weeks (younger) female mice (Figure 3). Similar to older mice, the younger female mice at 120 h p.i. had kidney uptake that was consistently higher for mutants, with 2A10-VH-Fc_GRLR_ showing the highest (7.85 ± 0.63 SUV_mean_, 29.87 ± 5.04%ID/g) (Figures S8 and S9; Table S7 and S8). The 2A10-VH-Fc_LALAPG_ exhibited elevated renal uptake in younger mice (4.46 ± 0.27 SUV_mean_; 17.10 ± 0.82%ID/g), while 2A10-VH-Fc_WT_ remained low across all groups (1.59 ± 0.14 SUV_mean_; 4.74 ± 0.61%ID/g in younger mice; 1.15 ± 0.18%ID/g in older females). Although the younger mice exhibited higher kidney uptake (SUV_mean_ and %ID/g) of the mutants compared with the older mice, the difference was not statistically significant (young vs old, 2A10-VH-Fc_LALAPG_: ns, *p* = 0.1421; 2A10-VH-Fc_GRLR_: ns, *p* = 0.8501). In contrast, both mutants demonstrated significantly higher kidney uptake compared with 2A10-VH-Fc_WT_ (*p* < 0.0001) in younger (15–16 weeks) mice, suggesting age has minimal impact on renal uptake of 2A10-VH-Fc mutants.

Tumor uptake did not show significant differences, remaining comparable across age groups (Figure 3E,F, Tables S4 and S8). At 120 h p.i., [^89^Zr]­Zr-2A10-VH-Fc_LALAPG_ reached 10.86 ± 0.78%ID/g in younger mice versus 13.78 ± 0.78%ID/g in older females. Similarly, [^89^Zr]­Zr-2A10-VH-Fc_GRLR_ showed 9.33 ± 1.79%ID/g in younger mice and 10.46 ± 1.34%ID/g in older mice. The [^89^Zr]­Zr-2A10-VH-Fc_WT_ reached 7.97 ± 2.06%ID/g by 120 h in younger mice. Liver and spleen uptake remained significantly lower for both Fc mutants (*p* < 0.0001) compared with [^89^Zr]­Zr-2A10-VH-Fc_WT_, following the same trend observed in older mice.

### Gender Variable PET Imaging and Biodistribution of 2A10-VH-Fc Mutants

Given the superior *in vivo* stability of [^89^Zr]­Zr-2A10-VH-Fc_LALAPG_ compared with [^89^Zr]­Zr-2A10-VH-Fc_GRLR_, it was further evaluated to assess sex-based differences in biodistribution (Figure 3A,D). At 120 h p.i., male mice (18–19 weeks) showed significantly lower renal uptake with an SUV_mean_ of 2.42 ± 0.17 and %ID/g of 4.65 ± 0.74 than age-matched females (4.71 ± 0.26 SUV_mean_; 16.17 ± 0.45%ID/g, *p* < 0.0001) (Figure S10 and Table S9). Tumor uptake was slightly higher in male mice with an SUV_mean_ of 2.04 ± 0.65 and %ID/g of 13.03 ± 0.95 compared with females (1.69 ± 0.63 SUV_mean_; 13.78 ± 0.78%ID/g), but it was not significant (*p =* 0.3166).

Liver (7.50 ± 0.90 vs 4.27 ± 0.57%ID/g, *p* < 0.0020) and spleen (10.76 ± 2.10 vs 9.25 ± 0.90%ID/g, *p* < 0.2461) were also lower in males, with corresponding improvements in tumor-to-background (T/B) ratios (Figure S11 and Table S10). Taken together, Fc mutants successfully reduced off-target liver and spleen accumulation, enhancing T/B contrast, while females demonstrated elevated renal retention, suggesting a sex-specific influence on the renal handling of Fc mutants. No significant differences were observed with mouse strain or age, suggesting that renal uptake variability is primarily driven by sex rather than strain-dependent or age-related factors.

### Pan Applicability of 2A10-VH-Fc_LALAPG_ in Cancer Xenografts

The [^89^Zr]­Zr-2A10-VH-Fc_LALAPG_ mutant, which demonstrated higher tumor accumulation and reduced off-target distribution in HCT116 xenografts relative to those of [^89^Zr]­Zr-2A10-VH-Fc_WT_ and [^89^Zr]­Zr-2A10-VH-Fc_GRLR_, was further assessed in additional MSLN-positive xenograft models. Experimental variables, including cell passage, mouse age, and sex distribution, are detailed in Table S11. For the epidermoid carcinoma model, we selected A431-H9 and A431-G9 cells. Briefly, A431-G9 cells are engineered derivatives of the parental A431-H9 cell line that express a mutant form of MSLN that reduces MSLN shedding by approximately 80%.^28,29^ The phenotype of A431-G9 cells enabled consistent cell-based assays; corresponding results are provided in the Supporting Information (Figure S12). For the pancreatic cancer model, the AsPC-1 cell line was used, which expresses moderate endogenous levels of MSLN.^28^

#### MSLN Expression by Western Blot and Immunohistochemistry

MSLN expression was assessed by Western blot (WB) and immunohistochemistry (IHC) in HCT116, A431-H9, A431-G9, and AsPC-1 cell lines and corresponding xenografts (Figures 4 and S13). WB confirmed high MSLN expression in A431-G9 and A431-H9, moderate in AsPC-1 and HCT116, and absence in the HEK293T negative control. Notably, these expression patterns were highly consistent with IHC findings, reinforcing the reliability and biological relevance of the MSLN expression profile across platforms. A431-G9 tumors exhibited diffuse, moderate to strong membranous staining, while A431-H9 tumors demonstrated diffuse membranous staining with variable intensity ranging from mild to strong depending on tumor region. Additionally, A431-H9 contained foci of strong intracytoplasmic staining in a morphologically distinct cell population, potentially representing a tumor subset or activated fibroblasts. AsPC-1 xenografts showed heterogeneous staining, with ∼75% of cells exhibiting mild-to-moderate intracytoplasmic staining and scattered cells displaying strong membranous or cytoplasmic staining. HCT116 tumors showed multifocal mild cytoplasmic staining, with ∼75% of cells demonstrating mild-to-moderate membranous staining and rare instances of strong expression. Minimal background staining in MSLN-negative and isotype controls confirmed antibody specificity.

**Figure 4:**
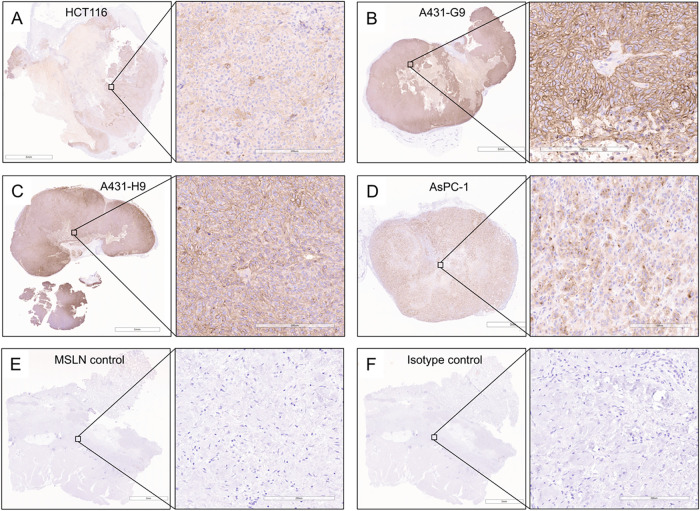
Immunohistochemistry images demonstrating MSLN expression in HCT116, A431-G9, A431-H9, and AsPC-1 xenograft tumor sections (A–D). Nonspecific staining was assessed using an isotype control primary antibody for the respective xenograft. MSLN control (E) and IgG2B kappa isotype control (F).

#### Validation of VH-Fc Fusion Proteins in Murine Models of Multiple Cancer Models

Tumor uptake remained comparable across sexes, ages, and tumor models. In A431-G9 xenografts, tumor uptake reached 16.00 ± 3.98%ID/g in young males (10–11 weeks), 17.20 ± 10.83%ID/g in young females, and 16.51 ± 4.39%ID/g in older males (18–19 weeks; Figure 5 and Table S12). A431-H9 showed similar tumor accumulation: 19.57 ± 10.07%ID/g in young males, 11.67 ± 1.09%ID/g in young females, and 11.29 ± 2.35%ID/g in older males (Figures 5, S14–S16; Tables S13–S15). Similar to the HCT116 models, female mice exhibited significantly higher kidney uptake across all tumor models. In A431-G9 xenografts, *ex vivo* kidney uptake at 120 h p.i. was 10.14 ± 2.79%ID/g in young females (10–11 weeks) compared with young males (10–11 weeks, 4.37 ± 1.03%ID/g; *p* < 0.0082) and older males (18–19 weeks, 4.54 ± 0.47%ID/g; *p* < 0.0028). In A431-H9 tumor mice, females showed elevated renal retention of 9.63 ± 1.57%ID/g, while younger (4.81 ± 1.29%ID/g; *p* < 0.0032) and older (3.90 ± 1.15%ID/g; *p* < 0.0011) males had lower uptakes, respectively (Figure S17). The AsPC-1 (male, 18–19 weeks) had kidney uptake of 4.52 ± 0.28%ID/g at 120 h and the highest tumor uptake, reaching 21.22 ± 8.73%ID/g at 120 h (Figure S18; Tables S16 and S17). Liver and spleen uptake remained low across all tumor models, reflecting the favorable off-target profile of the Fc-engineered [^89^Zr]­Zr-2A10-VH-Fc_LALAPG_ and its ability to enhance the T/B contrast. These results confirm that sex, rather than age or tumor model, is a key factor influencing the renal handling of Fc mutants, with females consistently showing higher renal retention.

**Figure 5:**
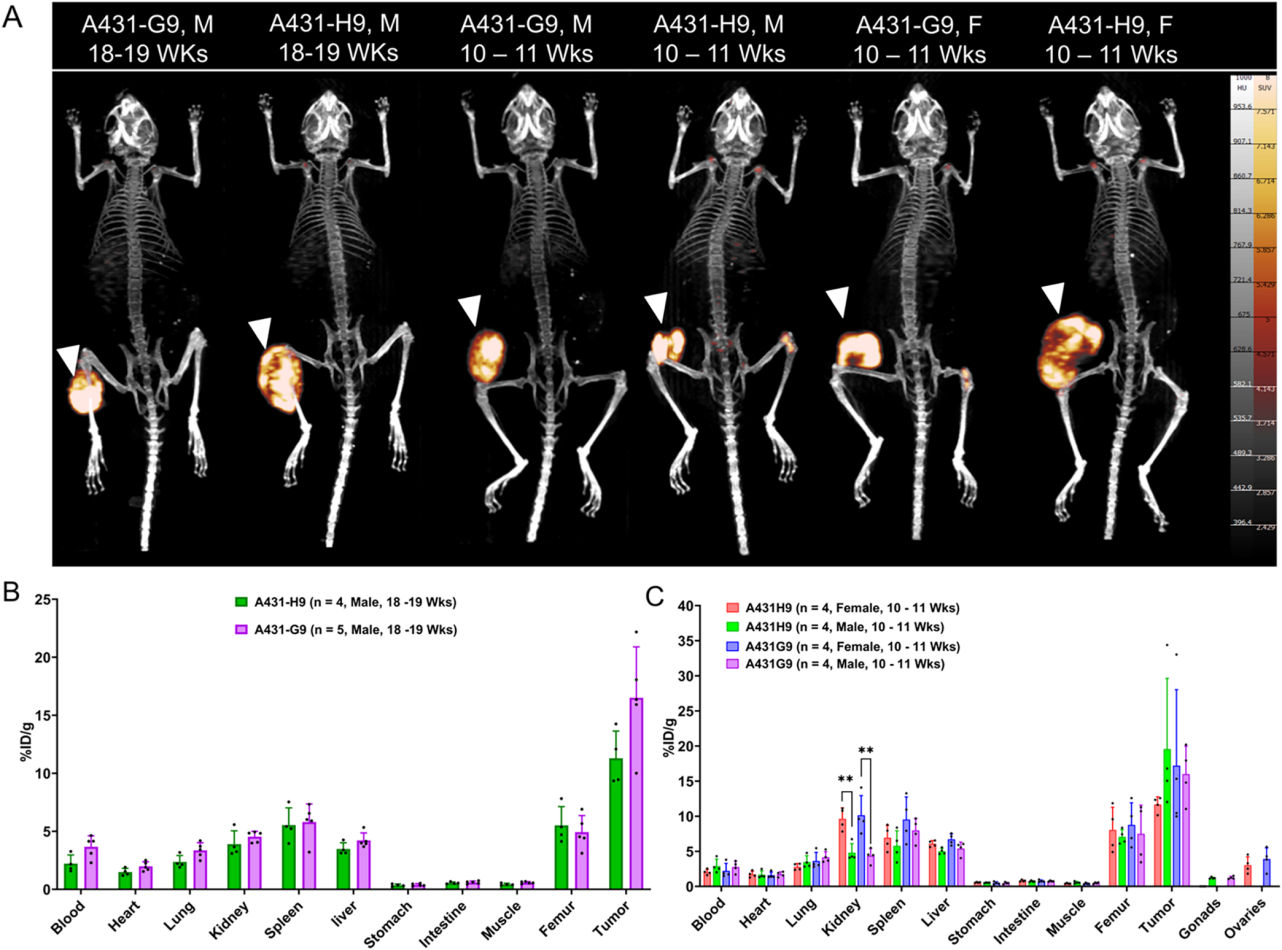
Representative MIP PET/CT images acquired at 120 h p.i. of [^89^Zr]­Zr-2A10-VH-Fc_LALAPG_ in respective male and female mice. White arrowheads indicate tumors (A). Biodistribution of [^89^Zr]­Zr-2A10-VH-Fc_LALAPG_ in A431-H9 and A431-G9 in older male mice (B) and younger male and female mice (C), at 120 h p.i. **p ≤ 0.01.

### Evaluation of Intratumoral Distribution by iQID Imaging and Histology Analyses

To explore 2A10-VH-Fc fusion proteins’ intratumoral distribution, HCT116 xenografts were collected 20 h p.i., sectioned consecutively, and analyzed by high-resolution iQID imaging with corresponding histology analyses. Tumor sections from VH-Fc fusion proteins were compared with nontargeting vector control Ab6-VH-Fc_WT_. All [^89^Zr]­Zr-2A10-VH-Fc agents showed higher and more tumor-localized uptake than the control, [^89^Zr]­Zr-Ab6-VH-Fc_WT_. The [^89^Zr]­Zr-2A10-VH-Fc_WT_ exhibited peripheral uptake with limited core penetration, whereas [^89^Zr]­Zr-2A10-VH-Fc_LALAPG_ achieved a more homogeneous and deeper distribution. The [^89^Zr]­Zr-2A10-VH-Fc_GRLR_ demonstrated intermediate characteristics, with improved core distribution compared with [^89^Zr]­Zr-2A10-VH-Fc_WT_ but not as uniform as [^89^Zr]­Zr-2A10-VH-Fc_LALAPG_. Minimal signal from vector control tumor supported MSLN-specific uptake. The iQID signal with high-density tumor regions was observed in H&E-stained sections, confirming that the radiotracer uptake corresponded to viable tumor areas and further validating the receptor-specific delivery of these agents (Figure 6).

**Figure 6:**
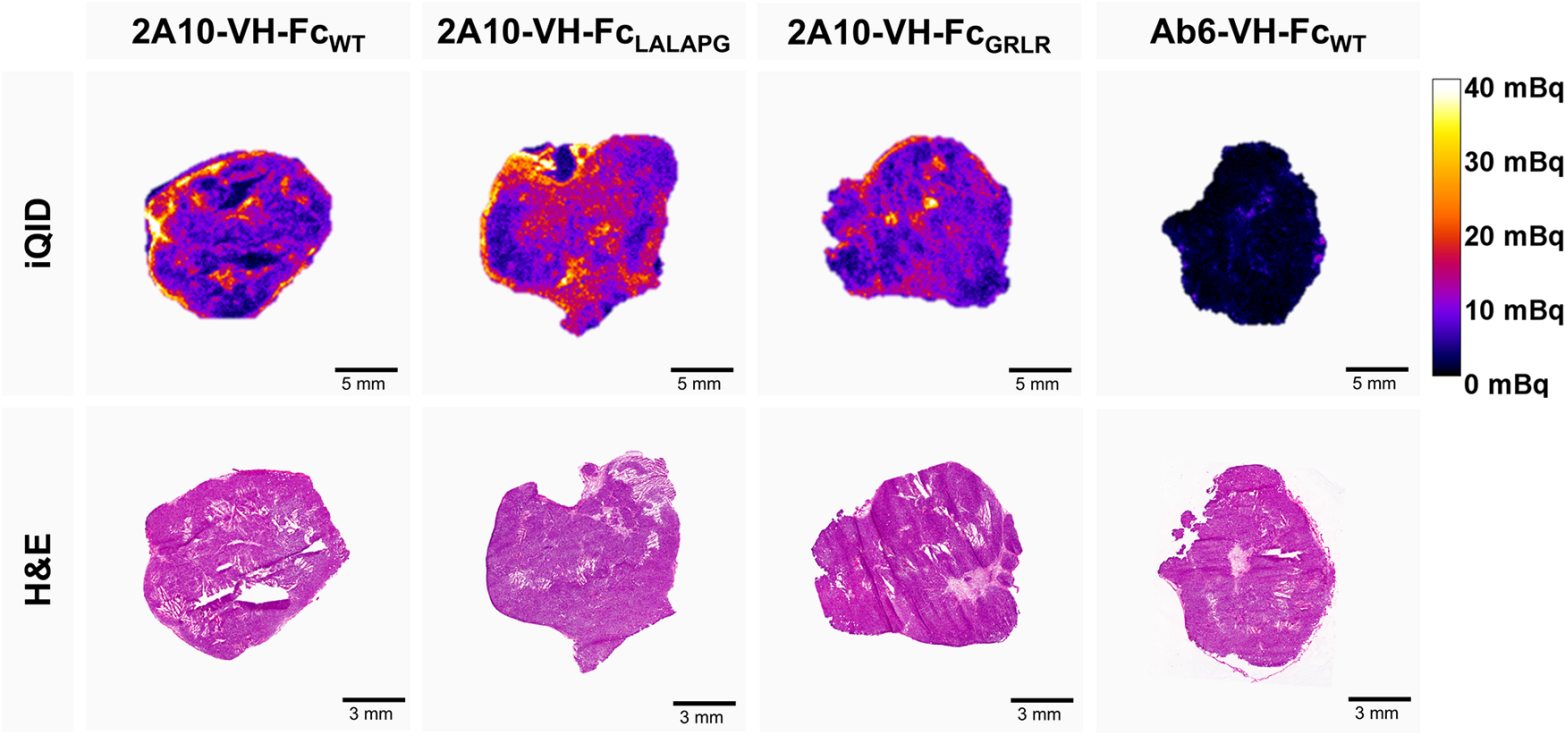
iQID imaging and histological correlation of [^89^Zr]­Zr-labeled VH-Fc fusion proteins in HCT116 tumor models (female; 18–19 weeks) at 20 h p.i. Top: intratumoral distribution of [^89^Zr]­Zr-labeled 2A10-VH-Fc_WT_, -LALAPG, -GRLR, and vector control Ab6-VH-Fc_WT_ with individual sections showing distribution uniformities (activity scale: 0–40 mBq). Bottom: corresponding H&E-stained sections illustrating uptake colocalization with viable tumor regions, reinforcing receptor-specific distribution.

## Discussion

Previously, we introduced next-generation VH-Fc fusion proteins targeting MSLN that offered enhanced tumor uptake, penetration, and plasma half-lives compared with conventional monoclonal antibodies.^11^ However, VH-Fc variants were associated with Fc-mediated sequestration in Fc-rich organs like the liver and spleen. In both our previous and current studies, we utilized NOD-Prkdc^em26Cd52^Il2rg^em26Cd22^/NjuCrl (NCG) mice, which lack mature T, B, and NK cells but retain myeloid populations (monocytes, neutrophils, macrophages, and dendritic cells) within bone marrow, liver, and spleen, which express high levels of FcRs. The absence of endogenous IgG resulting from B-cell dysfunction renders exogenously administered antibodies susceptible to nonspecific Fc-mediated uptake in these tissues.^30^ Sharma et al. demonstrated that the host immunodeficiency significantly influences antibody biodistribution, with NSG mice (similar to NCG) showing increased hepatic and splenic retention due to the lack of endogenous IgG, whereas Nu/Nu mice with intact IgG show reduced deposition.^30^ Although less immunodeficient strains are preferred for evaluating antibody-based therapies, their competent NK and other innate immune functions may exhibit xenoreactivity, adversely affecting cancer-derived xenograft (CDX) and patient-derived xenograft (PDX) engraftment. In our previous study, an irrelevant IgG1-Fc-blocking agent enhanced the tumor accumulation of [^89^Zr]­Zr-2A10-VH-Fc_WT_ and [^89^Zr]­Zr-m912 anti-MSLN antibodies in NCG xenograft.^11^ However, the efficacy of utilizing the Fc silencing strategy to improve the imaging contrast in the clinic remains to be explored, given that most patients will not be as immunocompromised as NCG mice.

In contrast, Fc silencing may reduce FcγR activation and Fc-mediated toxicity. Strohl et al. highlighted that Fc-engineering can modulate antibody PK and limit immune-related adverse effects, collectively enhancing therapeutic performance.^31^ Schlothauer et al. reported that FcγR silencing improves antibody stability and prolongs serum half-lives.^19^ Chan and Carter further underscore the role of Fc-interactions in autoimmune and inflammatory diseases to optimize immune modulation.^32^ Overall, Fc-engineering has become crucial to improving PK and distribution of radio-immunoconjugates, by modulating interaction with the FcγRs and the neonatal Fc receptor (FcRn).^13,33,34^

To mitigate Fc-mediated challenges, we engineered 2A10-VH-Fc mutants by introducing GRLR and LALAPG mutations to eliminate FcγR binding, thereby minimizing immune clearance and enhancing tumor targeting.^14,35^ Both mutants displayed significant improvement in distribution compared with wild-type, showing significantly reduced liver and spleen uptake, consistent with prior Fc-blocking studies linking FcγR interactions to enhance hepatic and splenic sequestration. These findings align with Mangeat et al., who demonstrated LALAPG-modified IgGs exhibit >4-fold lower liver retention with preserved tumor accumulation.^14^ Tumor accumulation was significantly increased for both mutants: [^89^Zr]­Zr-2A10-VH-Fc_LALAPG_ by 3.3-fold (*p* < 0.0001) and [^89^Zr]­Zr-2A10-VH-Fc_GRLR_ by 2.5-fold (*p* < 0.0001), relative to wild-type, [^89^Zr]­Zr-2A10-VH-Fc_WT_ (Figure 3A,E). [^89^Zr]­Zr-2A10-VH-Fc_LALAPG_ was further evaluated as a pan-targeted PET agent as it demonstrated robust and tumor-specific localization, favorable PK, and reduced off-target accumulation compared with 2A10-VH-Fc_WT_ and VH-Fc_GRLR_. Furthermore, it demonstrated selective targeting across MSLN-expressing xenografts (A431-G9, A431-H9, HCT116, and AsPC-1), consistently yielding a strong tumor signal even in HCT116 tumors with low-to-moderate MSLN expression by IHC and WB.

However, PET and biodistribution revealed higher kidney deposition for mutants than the [^89^Zr]­Zr-2A10-VH-Fc_WT_. Further investigation indicated the mutants were more susceptible to degradation, leading to kidney clearance, particularly in female mice. Female mice exhibited significantly higher kidney retention for all VH-Fc fusion proteins, which persisted across multiple tumor models and mouse strains, but the differences were greater in the mutants than in VH-Fc_WT_. SEC-HPLC of urine detected minor degradation, with new peaks that did not correspond to intact 2A10-VH-Fc or 2A10-VH domain, suggesting metabolic byproducts rather than structural instability.^36,37^

Both wild-type and mutant proteins exhibited similar folding by SEC and comparable binding affinities by BLI (Figure 1C,D). As Fc mutations typically do not alter overall structure,^38^ the differential renal deposition is likely attributable to changes in molecular polarity and surface charge. The GRLR mutation introduces a positively charged arginine, potentially enhancing electrostatic interactions with the negatively charged glomerular basement membrane, thereby explaining its higher renal accumulation compared with the LALAPG.^39^ Consistent with this, mutant tracers preferentially localized to the renal cortex (Figure S19), supporting electrostatic-attraction-driven glomerular retention. Strategies to mitigate such retention include modulating surface charge or other biophysical properties, for example, by altering linkers or chelators, or by incorporating negatively charged amino acids.

However, this glomerular retention mechanism cannot explain why VH-Fc variants show minimal renal deposition in male mice, prompting an alternative hypothesis: the enrichment of radioactivity within the renal cortex might be due to internalization or trapping of catabolized antibody fragments in proximal tubular cells following filtration. Although VH-Fc fusion proteins (∼80 kDa) are above the glomerular filtration cutoff, partial catabolism could generate fragments capable of filtration, as observed by urine SEC-HPLC analysis (Figure S5). In this sense, the sex-dependent differences in expression of catabolic enzymes and tubular reabsorption receptors may contribute to the greater cortical accumulation observed in female mice. These observations align with findings by McDonough et al. (2024), highlighting sex-dependent expression patterns of renal transporters, such as OATs, SGLTs, and NHE3, which may contribute to the higher renal uptake in females.^40^ If this tubular reabsorption mechanism is dominant, we can infuse the polypeptide-based plasma expander, gelofusine, to decrease renal accumulation.^41^ Nevertheless, our results showed that the mutants significantly outperformed the wild-type 2A10-VH-Fc fusion protein, providing promising anti-MSLN targeting agents.^36,37,42^

## Conclusion

In summary, this study demonstrates that Fc-engineering of anti-MSLN VH-Fc fusion proteins, particularly those bearing the LALAPG mutation, provides significant improvements in tumor targeting and biodistribution. Selective modulation of Fc silencing to reduce FcγR interactions improved tumor-specific accumulation and provided favorable tumor-to-background contrast. These promising results support the continued development of these Fc-engineered VH-Fc mutants for diagnostic and therapeutic applications of MSLN-expressing tumors. Additionally, the observed sex-related difference highlights the importance of incorporating both sexes in the preclinical evaluation. The 2A10-VH-Fc_LALAPG_ agent stands out as a lead candidate, with a compelling profile for advancing its development into more effective PET-guided therapeutic platforms.

## Experimental Section

Detailed experimental procedures are provided in the Supporting Information.

All animal studies were conducted in accordance with the guidelines of the Institutional Animal Care and Use Committee (IACUC) of the University of Pittsburgh and approved by the Division of Laboratory Animal Resources (DLAR) under protocol (24024565).

All work involving biohazardous, radioactive, and chemical materials was conducted by trained and authorized personnel in compliance with approved protocols from the University of Pittsburgh’s Institutional Biosafety Committee, Environmental Health and Safety, and Radiation Safety Office.

### Statistical Analysis

Statistical analysis was performed using the GraphPad online unpaired *t* test calculator (two-tailed). Data are presented as mean ± standard deviation (SD), and group comparisons were made by using two-tailed unpaired *t* tests. A *p*-value of less than 0.05 was considered statistically significant. The following thresholds were used to indicate levels of significance: *p < 0.05, **p < 0.01, ***p < 0.001, and ****p ≤ 0.0001.

## Supplementary Material



## Notes

Detailed materials and methods for the experimental section; additional figures and tables for LC-MS characterization of VH-Fc fusion proteins, stabilities, PET imaging analysis, radiolabeling summaries, cell line information, Western blot analysis, and iQID images (PDF)
