# Supplementary material for: Fc-Engineering Improves PET Imaging of Anti-Mesothelin VH-Fc across Multiple Tumor Mouse Models and Reveals Sex-Specific Renal Clearance
Source: Bioconjug Chem. 2025 Dec 24;37(1):192–202. doi: 10.1021/acs.bioconjchem.5c00591 (PMC12828721; doi:10.1021/acs.bioconjchem.5c00591)
Supplement: Supplementary file 1 [file bc5c00591_si_001.pdf]

Supporting Information

Fc engineering Improves PET Imaging of Anti-Mesothelin VH-Fc Across Multiple Tumor Mouse Models and Reveals Sex-Specific Renal Clearance

Abhinav Bhise[†, §, #], Xiaojie Chu[‡, #], Anders Josefsson[†, §], Angel G. Cortez[§], George Diehl[†, §], Lora H. Rigatti[§, ¶], Hyun Jung Park[‖], Jessie R. Nedrow[†, §, **], Wei Li[‡, **]

[†]Department of Radiology, University of Pittsburgh School of Medicine, Pittsburgh, PA, USA

[§]Hillman Cancer Center, University of Pittsburgh School of Medicine, Pittsburgh, PA, USA

[‡]Center for Antibody Therapeutics, Division of Infectious Diseases, Department of Medicine, University of Pittsburgh, School of Medicine, Pittsburgh, PA, USA

[¶]Division of Laboratory Animal Resources, University of Pittsburgh School of Medicine, Pittsburgh, PA, USA

[‖]Department of Human Genetics, University of Pittsburgh, Pittsburgh, PA, USA

[#]Authors contributed equally

**Co-corresponding authors

Corresponding Authors:

Jessie R. Nedrow, Ph.D.

5117 Centre Avenue, Suite G. 17b, Pittsburgh, PA 15213, USA

Phone Office: +1 (412) 623-7239; Email: nedrowj@upmc.edu

Wei Li, Ph.D.

S843 Scaife Hall, 3550 Terrace Street, Pittsburgh, PA 15261, USA

Phone Office: +1 (412) 383-4703; Email: liwei171@pitt.edu

EXPERIMENTAL SECTION

General information

All reagents and solvents were obtained from Sigma–Aldrich (St. Louis, MO, USA), unless otherwise noted. The p-SCN-Bn-DFO chelator was acquired from Macrocyclics, Inc. (Dallas, TX, USA), while [^{89}Zr]Zr-oxalate was sourced from the University of Wisconsin (Madison, WI, USA) or Washington University (St. Louis, MO, USA). The NCG mice (Strain code 572) were obtained from Charles River Laboratories, Inc (Wilmington, MA, USA). The HCT116 human colorectal cancer cell line and AsPC1 human pancreatic ductal adenocarcinoma cell line were obtained from the American Type Culture Collection (ATCC, Manassas, VA, USA). The HCT116 and AsPC1 cells were cultured in McCoy's media supplemented with 10% fetal bovine serum (FBS) and RPMI (10% FBS) respectively under standard conditions of a 5% CO2 atmosphere at 37 °C. MSLN inhibited cells A431-G9 and A431-H9 were generously obtained from (National Institutes of Health, Prof. Pastan Ira) and were supplemented with DMEM (10% FBS).

Generation anti-MSLN VH-Fc mutants to optimize imaging application

Human VH-Fc domain mutants GRLR (G236R, L328R) and LALAPG (L234A, L235A, P329G) were generated using a site-directed mutagenesis Kit (Agilent Technologies, Santa Clara, CA, USA). Recombinant VH-Fc mutant antibodies were expressed and purified as previously described.[1] Briefly, the plasmid was transfected into Expi293 cells by polyethyleneimine (PEI) and incubated in 37°C incubator with 8% CO2 atmosphere and shaking at 125 rpm for 6 days. Cells were removed, and medium containing the VH-Fc mutants was incubated with Protein A resin (GenScript Biotech Corp., Piscataway, NJ, US). After washing the resin by PBS, the antibodies were eluted using pH 3.0 Citrate Buffer and neutralized with 1M Tris-HCl pH 8.0 solution. Finally, the antibodies were buffer exchanged to PBS and concentrated using 30 kDa Amicon Ultra centrifuge filter (MilliporeSigma, Burlington, MA, USA).

Size-exclusion chromatography (SEC)

The structure and purity of the VH-Fc mutants were analyzed by Superdex 200 Increase 10/300 GL chromatography (GE Healthcare, Uppsala, Sweden) as previously described33. Standard proteins Ferritin, Aldolase, Conalbumin, Ovalbumin, Carbonic anhydrase and Ribonuclease were used for calibration. 200 μg filtered antibodies in 1 × DPBS (Dulbecco's phosphate-buffered saline, Gibco, Thermo Fisher Sci., Waltham, MA, USA)) were analyzed. Antibodies were eluted by DPBS buffer at a flow rate of 0.45 mL/min.

Enzyme-linked immunosorbent assay (ELISA)

In the ELISA procedure outlined, 2 μg/ml of human CD64 (hCD64, FcγRI), human CD32 (hCD32, FcγRII), human CD16(hCD16, FcγRIII), mouse CD16, and mouse CD64 recombinant protein were coated onto a 96-well high-binding plate and incubated at 4°C overnight. Plates were then blocked with 5%MPBS (non-fat milk in PBS) for 1 hour at 37°C after washing with PBST buffer three times. Next, binders were added to the plate with a 3-fold serial dilution and incubated at 37°C for another 1 hour. After washing, anti-human Fc antibody conjugated with horseradish peroxidase (anti-hFc-HRP) was added to the wells and incubated at 37°C for 1 hour to enable detection of bound target molecules. The color was developed by substrate solution, 3,3',5,5'-tetramethylbenzidine (TMB), and stopped by TMB stop buffer. The absorbance of each well was measured at 450 nm using a microplate reader.

Bio-Layer Interferometry (BLItz)

The avidity of 2A10-VH-Fc mutants were detected by biolayer interferometry BLItz (ForteBio, Menlo Park, CA, USA) as previously described.[2] Briefly, 33.3 μg/ml recombinant MSLN-biotin were coated onto the streptavidin biosensors for 2 min, different concentration of 2A10-VH-Fc null were used for association and monitored for 2 min. Antibody dissociation was monitored in DPBS

for 4min. DPBS was used to establish a baseline for 30s. MSLN-coated biosensors with DPBS served as reference control.

Immunohistochemistry (IHC)

Formalin-fixed, paraffin-embedded (FFPE) tissue sections were subjected to deparaffinization and rehydration following standard histological procedures. Antigen retrieval was carried out using an EDTA buffer (Cell Signaling Technology, Danvers, MA, USA). Automated immunostaining was performed on the Autostainer Plus platform (Dako, Agilent Technologies, Santa Clara, CA, USA) with Tris-buffered saline containing Tween-20 (TBST) as the rinse buffer (Cell Signaling Technology, Danvers, MA, USA). For primary antibody staining, the slides were incubated at room temperature with anti-mesothelin (Clone: MSLN/2131, Novus Biologicals, Centennial, CO, USA; Catalog# NBP2-79724) and IgG2b kappa isotype control (Clone: eBMG2b, Invitrogen, Waltham, MA, USA; Catalog# 14-4732-82), both at a dilution of 1:100. A Mouse-on-Mouse HRP-Polymer detection system (Biocare Medical, Pacheco, CA, USA) was used as the secondary reagent. Signal development was achieved using 3,3′-diaminobenzidine (DAB)+ substrate (Cell Signaling Technology), followed by counterstaining with hematoxylin (Cell Signaling Technology).

Bioconjugation and Radiolabeling

The anti-MSLN VH-Fc proteins underwent modification with DFO and subsequent radiolabeling with Zr-89 according to established protocols.[3] Briefly, VH-Fc proteins were subjected to buffer exchange using a conjugation buffer composed of 500 mmol $NaHCO_3$, 20 mmol Na_2CO_3, 1.5 mol NaCl, and 10 mmol EDTA, using a 30 kDa filter (Vivaspin® 6, Cytiva, Sweden). *p*-SCN-Bn-DFO was added at a molar ratio of 1:5 and allowed to incubate for 1 hour at 37 °C. The resulting conjugates were washed with PBS using a 30 kDa filter to remove any unreacted DFO. For radiolabeling, 20-25 µL of a solution containing 2A10-VH-Fc mutant (WT, GRLR, and LALAPG), vector control Ab6-VH-Fc_{WT}, or respective VH-domains in PBS (pH 7.4) was added to a vial containing Zr-89 (approximately 14.8 – 18.5 MBq) in 25 µL of 1M oxalic acid and 175 µL of 1M

HEPES buffer at pH 8. The resulting mixture was incubated at 37 °C for 60 minutes on a thermomixer. Radiolabeling of VH-Fc conjugates was confirmed via instant radio-TLC on silica gel plates (iTLC-SG) developed in a 10 mM EDTA solution. Subsequently, the purified radiolabeled VH-Fcs were isolated using centrifugal filter units with a 30 kDa molecular weight cutoff. The purity of the radioimmunoconjugates was assessed using radio-TLC with a plate reader and SEC-HPLC. Protein concentrations were determined using nanodrop (Thermo Fisher Sci., Waltham, MA, USA).

Cellular uptake

A431G9 cells (100,000 per well, n = 3) were seeded in 6-well plates and cultured in DMEM with 10% FBS. After washing with 2 mL PBS, fresh media (2 mL/well) was added, followed by 370 kBq (10 μCi) of [^{89}Zr]Zr-2A10-VH-Fc_{LALAPG} or [^{89}Zr]Zr-AB6-VH-Fc_{WT} in 10 μL. Cells were incubated for 1 hour at 37°C in a CO_2 incubator. Supernatants were collected in HPLC tubes, and cells were washed with 2 mL PBS before lysing with 2 mL of 0.5% SDS. Lysates were transferred to HPLC tubes for counting in a γ-well counter (PerkinElmer 2480 WIZARD^2 Automatic Gamma Counter, MA, USA).

Internalization Assay

A431-G9 cells (1.0 × 10^5 cells/well) were plated on 6-well plates and incubated for 24 hours (n = 3). The cells were washed twice with 1 mL PBS, followed by the addition of 1 mL growth media (DMEM + 10% FBS) and incubation at 37°C for 10 minutes. The [^{89}Zr]-Zr-2A10-VH-Fc (WT, GRLR and LALAPG, and Ab6-VH-Fc_{WT}) were added and incubated at 37°C for 1-, 2-, 4-, 16-, and 24-hours. Three aliquots of standards were saved at each time point for counting. After incubation, the radioactive media was aspirated, and the cells were washed with 2 mL ice-cold PBS. To remove surface-bound radioactivity, the cells were treated with 1 mL acid wash (pH 3) for 10 minutes and rinsed with an additional 1 mL acid wash. Cells were then lysed with 1 mL 0.5% SDS, and the fractions were collected to measure radioactivity using a γ-well counter. To account

for sample variability, radioactivity measured in internalization assays was normalized to total cell counts. Percent uptake obtained from the γ-well counter was multiplied by the corresponding cell number and divided by one million to calculate the final uptake as percent per 10^6 cells.

Western Blot

Cell lysates were prepared from MSLN-positive cell lines (A431-G9 , A431-H9, AsPC-1, and HCT116) and MSLN-negative cells (HEK293T) cells using RIPA Buffer (ThermoFisher Scientific , Waltham , USA) supplemented with 1% protease inhibitor (Thermo fisher Scientific , Waltham , MA). Total protein concentrations were quantified using the BCA Assay kit (Thermo Fisher Scientific, Waltham, MA, USA; Cat. No. 23225). Equal volumes of lysates corresponding to 10 µg of total protein (A431-G9 and A431-H9) or 40 µg (HCT116, AsPC-1, and HEK293T) were resolved on Mini-PROTEAN TGX precast gel (Bio-Rad Laboratories, Hercules, CA, USA; Cat. No. 4561094) at 200 V for 30 minutes. Proteins were transferred to 0.2 µm nitrocellulose membrane (Bio-Rad Laboratories, Hercules, CA, USA; Cat. No. 1620112). The membrane was blocked with 5% dry fat skim milk in 0.05% PBST (phosphate-buffered saline with Tween 20) for 1 h at ambient temperature. Subsequently, membranes were incubated overnight at 4 °C with primary antibodies diluted in blocking buffer: mouse monoclonal anti-MSLN antibody (1:2000; Rockland Immunochemicals, Limerick, PA, USA; Cat. No. 200-301-A88) and mouse anti-human vinculin monoclonal antibody (1:2000; Bio-Rad Laboratories, Hercules, CA, USA; Cat. No. MCA465GA). After washing, membranes were incubated with horseradish peroxidase (HRP)-conjugated anti-mouse IgG secondary antibody (1:5000; Cell Signaling Technology, Danvers, MA, USA; Cat. No. 7076) for 1 h at room temperature. Protein bands were visualized using enhanced chemiluminescence (ECL) detection reagents mixed at a 1:1 ratio (Bio-Rad Laboratories, Hercules, CA, USA; Cat. No. 170-5060), and signals were captured using the ChemiDoc MP imaging system. Quantification was performed using Image Lab software (Bio-Rad Laboratories). MSLN signal intensity in each lane was normalized to its corresponding vinculin loading control.

***In vitro* and *In vivo* stability**

Serum stability was assessed by incubating approximately 3.7 MBq of the radioimmunoconjugate [^{89}Zr]-Zr-2A10-VH-Fc (WT, GRLR and LALAPG) in PBS or human serum (HS) at ambient temperature and 37 °C respectively. Samples were withdrawn using a micropipette at 24, 48, and 72-hours post addition of PBS or HS and spotted on iTLC-SG to elute in 10 mM EDTA solution. The *in vitro* stability studies were performed in duplicate. For *in vivo* stability, approximately (1.66 – 3.3 MBq) of radioimmunoconjugates were administered through intravenous injection (i.v.) to NCG mice (n = 3). After 90 minutes post injection (p.i.), mice were euthanized to harvest the blood and kidneys and placed in an ice bath. The *in vivo* stability was determined by directly loading approximately 100 µL of blood on a pre-activated PD10 column and eluting with 20 mL of PBS (pH=7.4). The radioactivity in the collected fractions was measured using a γ-well counter. To determine the stability in the kidneys, the organs were homogenized using Cell strainer (40 µm Nylon, FALCON) with addition of PBS (200 µL) in ice cold conditions. Further, the homogenized organs were vortexed and centrifuged at 10,000 rpm for 5 minutes. The supernatant (100 µL) was loaded on the pre-activated PD10 column, and the same protocol was followed as described for the blood. The PD10 column was activated by washing with PBS (25 mL) before loading the samples.

Urine metabolization

In normal non-tumor bearing NCG mice (male, 8 weeks old, n = 3) radiolabeled mutated VH-Fc proteins were administered (13.69 – 16.87 MBq) and at 90 minutes p.i. mice were euthanized to collect urines. Followed by centrifugation and membrane filtration (Millex® – GV 0.22 µm, Merck Millipore Ltd.), urine was injected in Agilent Bio Sec-3 (4.6 × 150 mm, 3µm, 150Å) column to collect fractions at 0.4 mL/min. Counts were evaluated using γ-well counter and retention times were compared with the respective intact proteins.

Animal Models

All animal studies were conducted in accordance with the guidelines of the Institutional Animal Care and Use Committee (IACUC) of the University of Pittsburgh and approved by the Division of Laboratory Animal Resources (DLAR) under protocol (24024565). Tumor models (HCT116, A431-G9, A431-H9, and AsPC1) were developed by subcutaneous injection of 1 × 10^6 cells in PBS:Matrigel (1:1), which were routinely screened for mycoplasma contamination and found to be negative. Studies were conducted when tumors reached an approximate size of 100–200 mm^3.

PET/CT imaging and Biodistribution studies

PET-imaging experiments were conducted in NCG mice (female and male, 20−30 g, 14−18 weeks) with HCT116 tumors using an Inveon small animal micro-PET/CT system (Siemens Molecular Imaging, Knoxville, TN, USA), following previously established protocols.[2] The mice received intravenous (i.v.) injections of the radioimmunoconjugates (see Table 1) and were subsequently imaged at 90 minutes, 18/24, 48, and 120 hours. Imaging parameters included: 10 minutes PET acquisition time, employing 2DFPB for standard uptake value (SUV) calculations and OSEM3D for PET/CT reconstruction algorithms. CT-based attenuation correction was applied, with CT exposure settings at 80 kV, 500 µA, 145 ms exposure time, 220° rotation with 120 steps, low magnification, and bin 4 × 4. CT reconstruction utilized the Feldkamp algorithm with Shepp-Logan reconstruction filter, down sample factor 2, and image voxel sizes of x = 196.43 µm, y = 196.43 µm, z = 196.43 µm. PET/CT images were exported as DICOM files via VivoQuant Version 4.0patch3 (Invicro, Needham, MA, USA). Volumes of interest (VOIs) were delineated by CT for organs including the tumor, heart, vena cava (blood), muscle, marrow, kidneys, and liver. Muscle SUV's values below 0.1 were normalized to 0.1. The radioimmunoconjugates uptake in normal tissues and tumors was quantified as SUV_{mean} ± standard deviation. After PET imaging, mice were euthanized at 5-days p.i., and the following organs were collected: blood, heart, lungs, kidneys, spleen, liver, stomach (with contents), intestines (with contents), muscle, bone (femur with marrow), and tumors. These organs were then weighed and analyzed using an automatic γ-

well counter (PerkinElmer 2480 WIZARD[2]). The percentage of injected dose per gram (%ID/g) was determined by converting the decay corrected injected radioactivity to counts per minute (CPMs), considering the efficiency of Zr-89 radioisotope.

Ex Vivo Autoradiographic Imaging Using the iQID-Camera System

The iQID-camera system (QScint Imaging Solutions, Tucson, AZ, USA) was used to image the intra-tumoral distribution of [^{89}Zr]Zr-labeled VH-Fc fusion proteins (WT, LALAPG, and GRLR) 18-20 hours p.i. The HCT116 tumors (Female, 15 – 16 weeks), A431-G9 (Male, 18 – 19 weeks), A431-H9 (Male, 18 – 19 weeks), AsPC1 (Male, 18 – 19 weeks) were used as representative images (n=1). The harvested tumors were immediately harvested after sacrifice and embedded in optimal cutting temperature (OCT) resin and flash frozen. The frozen tissues were cut in 12-µm thick sections using a TN50 (Tanner scientific, Sarasota, FL, USA) cryostat and placed on a scintillator sheet QSCINT-02-0001 (QScint Imaging Solutions, Tucson, AZ, USA) for imaging. The samples were imaged in the iQID-camera using 40 frames per second (fps) and exposure times ranging between 24 hours. Images were processed using MATLAB R2023a (MathWorks Inc., Natick, MA, USA) and ImageJ2 v2.9.0/1.53t (National Institutes of Health, Bethesda, MD, USA) for image analysis.

Table S1. Summary of [^{89}Zr]Zr-labeled VH-Fc proteins injected into tumor models						
[^{89}Zr]Zr-labeled PET agents	Activity administered		Protein amount (µg/100 µL)	Molar activity (MBq/µmol)	Radio-labeling Efficiency	Radio-Purity
	MBq	µCi				
2A10-VH-Fc_{WT}	1.6 – 2.2	43 – 59	20.0 – 25.0	1.47 ± 0.3	≥95%	≥99%
2A10-VH-Fc_{LALAPG}	1.5 – 4.0	40 – 107	20.0 – 25.0	1.14 ± 0.3		
2A10-VH-Fc_{GRLR}	1.6 – 1.9	44 – 51	16.2 – 25.0	1.44 ± 0.4		

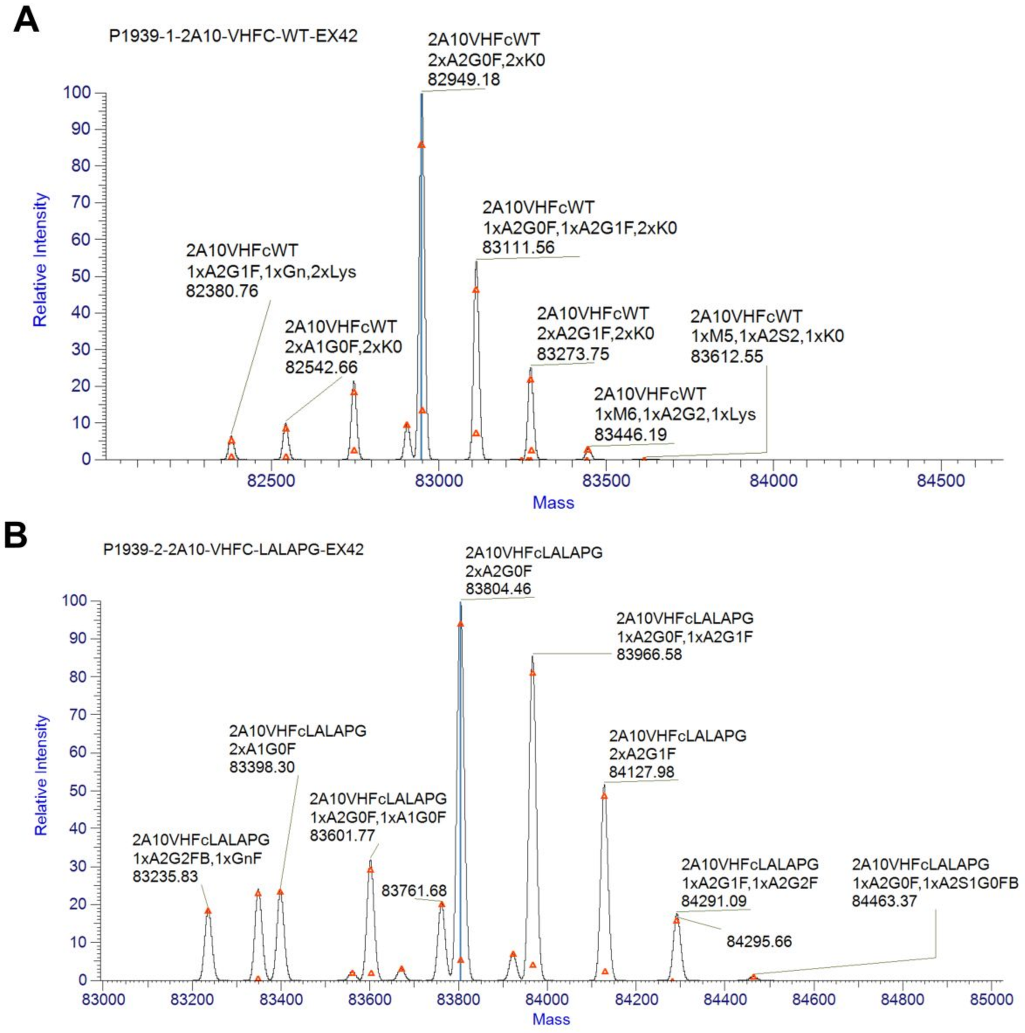

Figure S1: Deconvoluted Spectrum from Intact protein analysis of native 2A10-VH-Fc_{WT} and LALAPG. (A) Deconvoluted Spectrum of 2A10-VH-Fc_{WT} to the MW range from 80 kDa to 85kDa showing the glycan forms detected. K0 = C-terminus K removed form; 1 × Lys = adding an extra Lys at the C-terminus. This is commonly known as C-terminal lysine clipping of IgG1. (B) Deconvoluted Spectrum of 2A10-VH-Fc_{LALAPG} to the MW range from 83 kDa to 85 kDa showing the glycan forms detected.

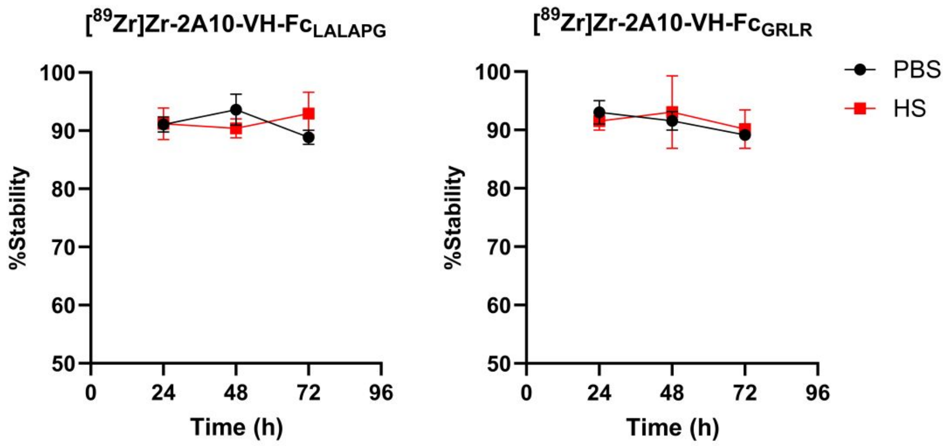

Figure S2: *In vitro* benchtop percent stability of zirconium-89 labeled LALAPG and GRLR mutant in phosphate buffered saline (PBS) and human serum (HS) at 24, 48, and 72h time points (n = 2).

**Table S2. *In vitro* stability studies of [^{89}Zr]Zr-labeled VH-Fc proteins**				
	**[^{89}Zr]Zr-2A10-VH-Fc$_{LALAPG}$**		**[^{89}Zr]Zr-2A10-VH-Fc$_{GRLR}$**	
**Time (h)**	**PBS**	**HS**	**PBS**	**HS**
24	91.1 ± 1.3%	91.2 ± 2.7%	91.2 ± 2.7%	91.5 ± 1.5%
48	93.6 ± 2.6%	90.4 ± 1.6%	90.4 ± 1.6%	93.1 ± 6.2%
72	88.9 ± 1.2%	93.0 ± 3.7%	92.9 ± 3.6%	90.2 ± 3.3%

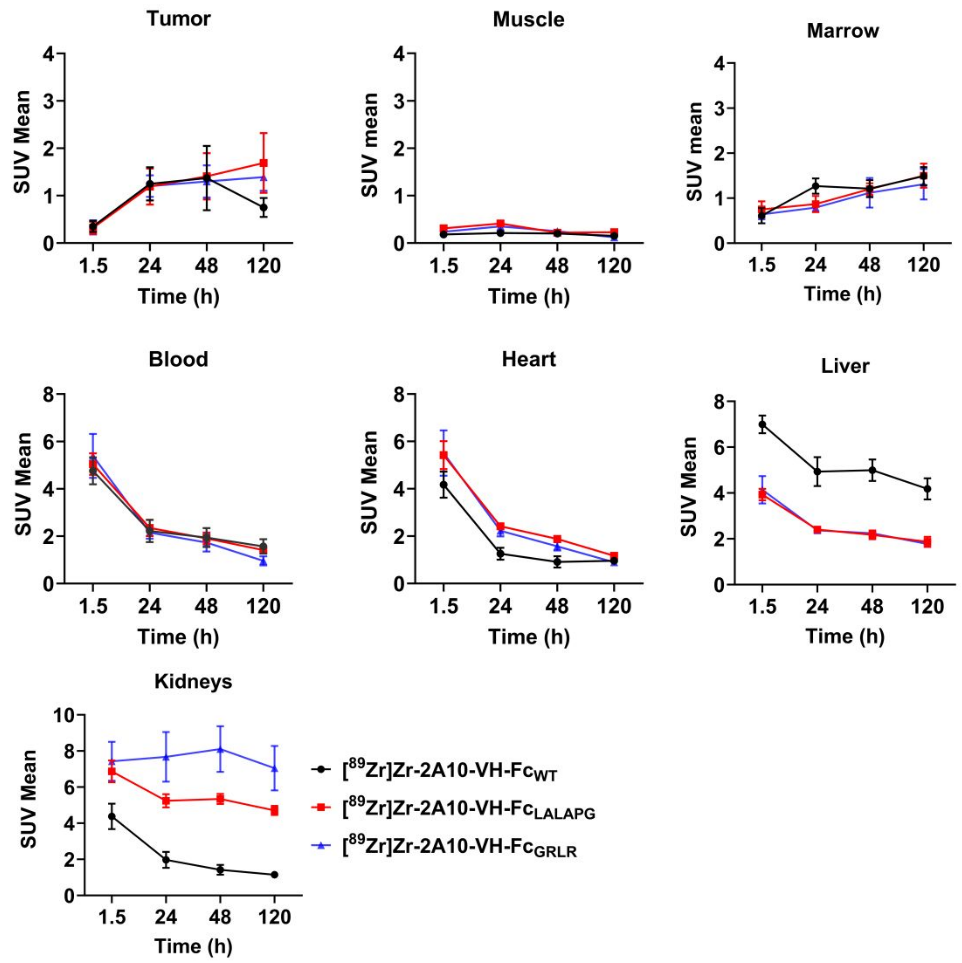

Figure S3: SUV_{mean} plot in HCT116 xenograft bearing female mice (18 – 19 weeks).

Table S3. SUV_{mean} values in HCT116 xenograft bearing female mice (18 – 19 weeks).				
**Organs**	**Time (h)**	**[^{89}Zr]Zr-2A10-VH-Fc_{WT}**	**[^{89}Zr]Zr-2A10-VH-Fc_{LALAPG}**	**[^{89}Zr]Zr-2A10-VH-Fc_{GRLR}**
**Tumor**	**1.5**	0.35 ± 0.12	0.32 ± 0.13	0.33 ± 0.15
	**24**	1.25 ± 0.35	1.19 ± 0.38	1.20 ± 0.23
	**48**	1.37 ± 0.68	1.41 ± 0.49	1.30 ± 0.34
	**120**	0.75 ± 0.20	1.69 ± 0.63	1.39 ± 0.29
**Blood**	**1.5**	4.76 ± 0.57	5.04 ± 0.46	5.39 ± 0.93
	**24**	2.22 ± 0.47	2.35 ± 0.35	2.15 ± 0.26
	**48**	1.94 ± 0.40	1.91 ± 0.25	1.73 ± 0.38
	**120**	1.57 ± 0.31	1.41 ± 0.14	0.96 ± 0.19
**Heart**	**1.5**	4.17 ± 0.55	5.42 ± 0.59	5.51 ± 0.96
	**24**	1.26 ± 0.25	2.42 ± 0.10	2.23 ± 0.24
	**48**	0.91 ± 0.24	1.88 ± 0.11	1.57 ± 0.18
	**120**	0.96 ± 0.12	1.17 ± 0.08	0.90 ± 0.16
**Muscle**	**1.5**	0.18 ± 0.04	0.31 ± 0.05	0.24 ± 0.07
	**24**	0.21 ± 0.06	0.41 ± 0.02	0.35 ± 0.10
	**48**	0.20 ± 0.02	0.22 ± 0.02	0.25 ± 0.06
	**120**	0.15 ± 0.06	0.23 ± 0.02	0.12 ± 0.02
**Marrow**	**1.5**	0.61 ± 0.17	0.75 ± 0.18	0.64 ± 0.11
	**24**	1.27 ± 0.17	0.87 ± 0.18	0.79 ± 0.10
	**48**	1.21 ± 0.19	1.20 ± 0.13	1.12 ± 0.33
	**120**	1.49 ± 0.20	1.50 ± 0.27	1.31 ± 0.34
**Liver**	**1.5**	6.99 ± 0.39	3.93 ± 0.25	4.14 ± 0.60
	**24**	4.93 ± 0.63	2.40 ± 0.11	2.37 ± 0.12
	**48**	4.99 ± 0.47	2.17 ± 0.19	2.24 ± 0.14
	**120**	4.18 ± 0.46	1.87 ± 0.22	1.77 ± 0.16

**Kidneys**	**1.5**	4.83 ± 0.71	6.87 ± 0.60	7.43 ± 1.08
	**24**	1.97 ± 0.44	5.24 ± 0.37	7.67 ± 1.38
	**48**	1.42 ± 0.27	5.34 ± 0.28	8.11 ± 1.27
	**120**	1.15 ± 0.18	4.71 ± 0.26	7.05 ± 1.23

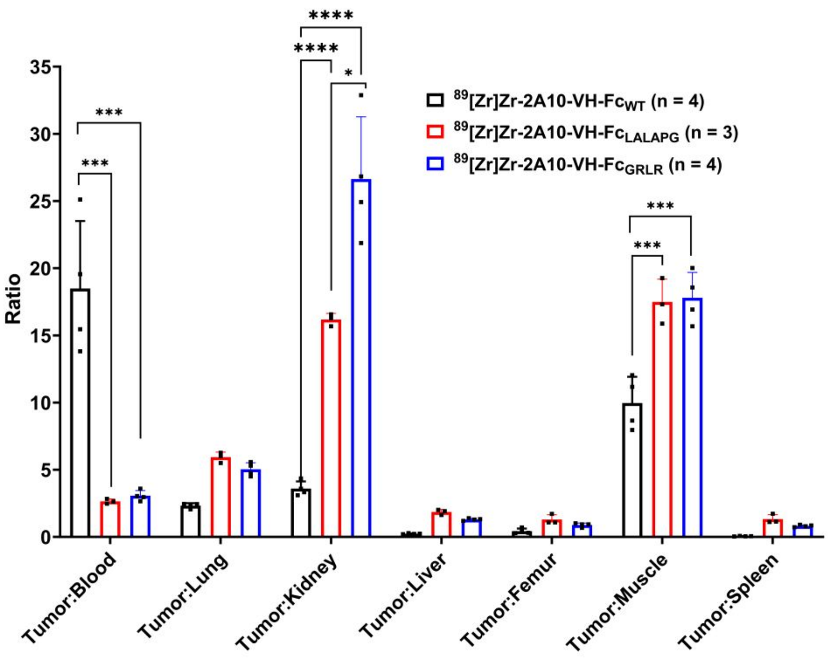

Figure S4: Bar plot demonstrating tumor-to-background ratios 5-days p.i. in HCT116 xenograft bearing mice (Female, 18 – 19 Weeks). ***p < 0.001 ****p < 0.0001.

**Table S4: Biodistribution (%ID/g) and tumor-to-background ratios 5-days p.i. in HCT116 xenograft bearing mice (Female, 18 – 19 weeks).**			
HCT116 (Female, 18 – 19 Wks)			
**Organs**	**[^{89}Zr]Zr-2A10-VH-Fc$_{WT}$ (n = 4)**	**[^{89}Zr]Zr-2A10-VH-Fc$_{LALAPG}$ (n = 3)**	**[^{89}Zr]Zr-2A10-VH-Fc$_{GRLR}$ (n = 4)**
Blood	1.43 ± 2.52	5.22 ± 0.30	2.71 ± 1.55
Heart	1.55 ± 0.16	2.68 ± 0.15	2.35 ± 0.28
Lung	2.23 ± 0.19	5.93 ± 0.39	5.03 ± 0.49
Kidney	3.59 ± 0.54	16.17 ± 0.46	26.62 ± 4.65
Spleen	94.95 ± 39.34	10.76 ± 2.12	12.98 ± 2.62
Liver	17.83 ± 2.37	7.50 ± 0.89	8.05 ± 0.90
Stomach	0.46 ± 0.13	0.58 ± 0.11	0.33 ± 0.08
Intestine	1.07 ± 0.10	0.88 ± 0.09	0.76 ± 0.16
Muscle	0.43 ± 0.11	0.79 ± 0.06	0.59 ± 0.08
Femur	10.79 ± 3.43	11.10 ± 2.29	11.92 ± 1.34
Tumor	4.17 ± 0.60	13.78 ± 0.78	10.46 ± 1.34
Ovaries	11.05 ± 2.94	6.42 ± 2.58	6.90 ± 0.70
**Tumor-to-background ratios**			
Tumor/Blood	36.66 ± 52.32	2.65 ± 0.18	3.17 ± 0.28
Tumor/Lung	2.32 ± 0.19	5.93 ± 0.39	5.03 ± 0.49
Tumor/Kidney	3.59 ± 0.54	16.17 ± 0.46	26.62 ± 4.65
Tumor/Liver	0.24 ± 0.03	1.85 ± 0.19	1.30 ± 0.08
Tumor/Muscle	0.43 ± 0.11	0.79 ± 0.06	0.59 ± 0.08

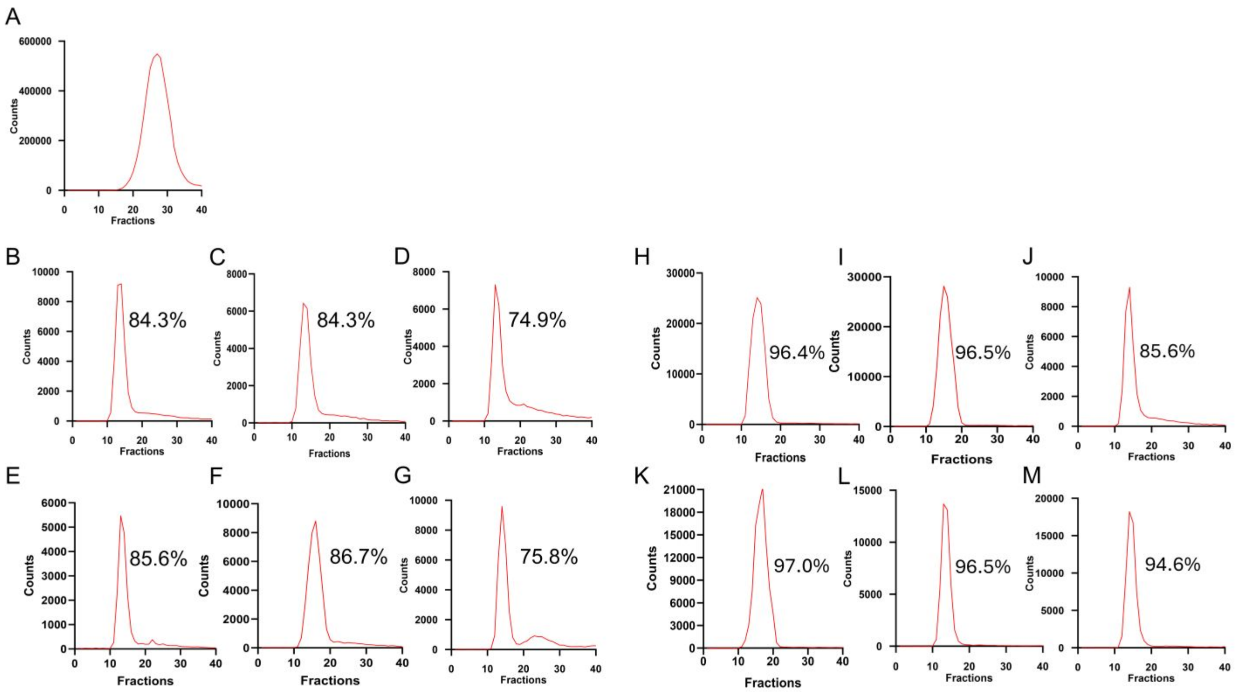

Figure S5: *In vivo* stability of zirconium-89 labeled GRLR in kidney (B-D) and blood (E-G), LALAPG Kidney (H-J) and Blood (K-M) 90 minutes p.i., and free zirconium-89 profile (A).

**Table S5. *In vivo* stability studies of $[^{89}Zr]Zr$-labeled VH-Fc proteins at 90 min p.i.**		
**Tissue**	**$[^{89}Zr]Zr$-2A10-VH-Fc_{LALAPG}**	**$[^{89}Zr]Zr$-2A10-VH-Fc_{GRLR}**
Blood	96.0 ± 1.3%	82.7 ± 6.0%
Kidney	92.8 ± 6.3%	81.2 ± 5.4%

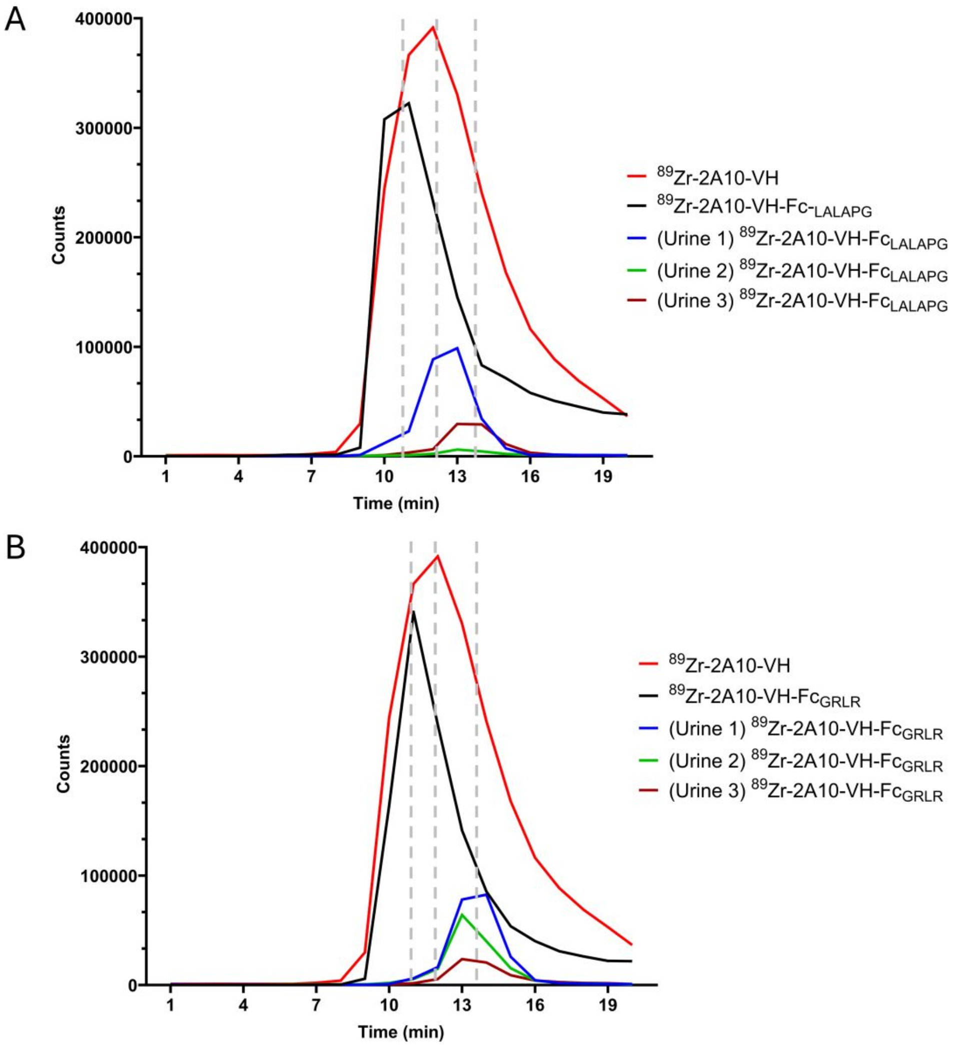

Figure S6: SEC-HPLC analysis of urine at 90 minutes p.i. confirms *in vivo* stability of [^{89}Zr]Zr-2A10-VH-Fc$_{LALAPG}$ (A) and VH-Fc$_{GRLR}$ (B). Most radioactivity corresponded to intact protein with minor metabolites present. Retention time of VH-Fc ~10.5 min, VH domain ~ 13.0 min, Urine ~13.5 – 14 min (n = 3).

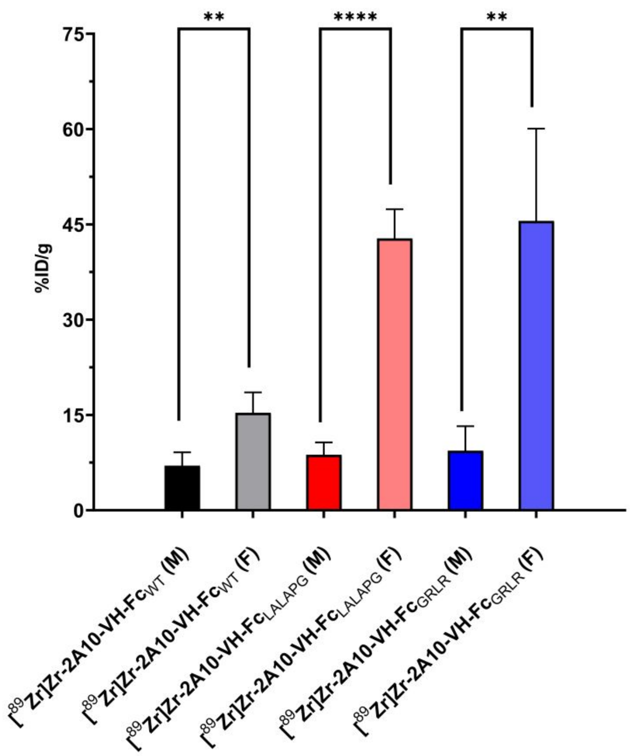

FigureS7: Kidney uptake of Fc-mutated VH-Fc mutants in CD1-IGS mice at 120 hours. Female mice showed significantly higher retention than male mice (n = 3). $^{**}p \leq 0.01$$^{****}p \leq 0.0001$.

**Table S6. Kidney uptake (%ID/g) of VH-Fc$_{WT}$ and Fc-mutated mutants in CD1-IGS mice at 120 hours (n = 3).**	
**Agents**	**CD1-IGS, Kidneys**
[^{89}Zr]Zr-2A10-VH-Fc$_{WT}$ (Male)	7.01 ± 2.1
[^{89}Zr]Zr-2A10-VH-Fc$_{WT}$ (Female)	15.34 ± 3.2
[^{89}Zr]Zr-2A10-VH-Fc$_{LALAPG}$ (Male)	8.73 ± 2.0
[^{89}Zr]Zr-2A10-VH-Fc$_{LALAPG}$ (Female)	42.78 ± 4.6
[^{89}Zr]Zr-2A10-VH-Fc$_{GRLR}$ (Male)	9.37 ± 3.9
[^{89}Zr]Zr-2A10-VH-Fc$_{GRLR}$ (Female)	45.56 ± 14.5

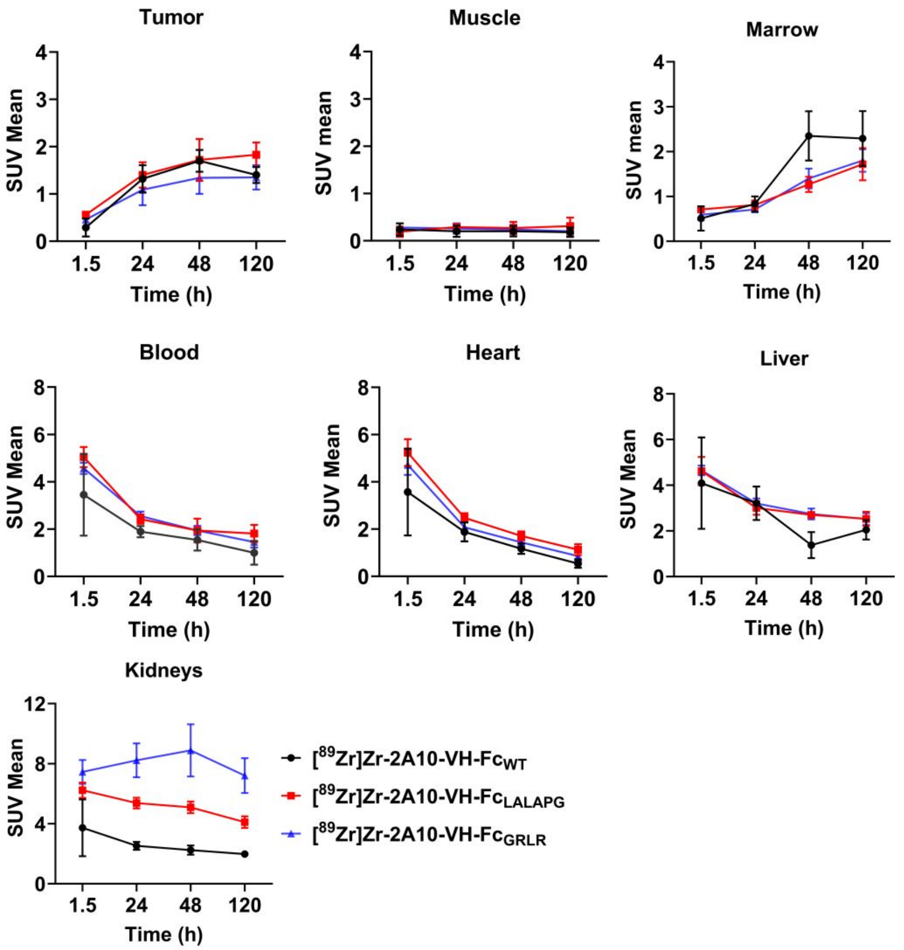

Figure S8: SUV_{mean} plot in HCT116 xenograft bearing female mice (15–16 weeks).

Table S7: SUV_{mean} values in HCT116 xenograft bearing female mice (15 – 16 weeks).				
**Organs**	**Time (h)**	**[^{89}Zr]Zr-2A10-VH-Fc_{WT}**	**[^{89}Zr]Zr-2A10-VH-Fc_{LALAPG}**	**[^{89}Zr]Zr-2A10-VH-Fc_{GRLR}**
**Tumor**	**1.5**	0.29 ± 0.19	0.56 ± 0.07	0.46 ± 0.08
	**24**	1.32 ± 0.29	1.40 ± 0.27	1.09 ± 0.33
	**48**	1.70 ± 0.23	1.72 ± 0.44	1.34 ± 0.34
	**120**	1.40 ± 0.17	1.83 ± 0.26	1.35 ± 0.26
**Blood**	**1.5**	3.45 ± 1.73	5.04 ± 0.43	4.57 ± 0.24
	**24**	1.89 ± 0.24	2.42 ± 0.20	2.55 ± 0.19
	**48**	1.54 ± 0.45	1.94 ± 0.50	1.94 ± 0.20
	**120**	1.00 ± 0.50	1.81 ± 0.37	1.45 ± 0.24
**Heart**	**1.5**	3.57 ± 1.84	5.24 ± 0.57	4.72 ± 0.43
	**24**	1.88 ± 0.40	2.48 ± 0.20	2.08 ± 0.21
	**48**	1.17 ± 0.22	1.71 ± 0.19	1.44 ± 0.19
	**120**	0.54 ± 0.18	1.13 ± 0.23	0.86 ± 0.19
**Muscle**	**1.5**	0.24 ± 0.13	0.19 ± 0.11	0.28 ± 0.08
	**24**	0.20 ± 0.12	0.29 ± 0.06	0.26 ± 0.11
	**48**	0.20 ± 0.11	0.27 ± 0.13	0.24 ± 0.10
	**120**	0.18 ± 0.10	0.31 ± 0.18	0.20 ± 0.12
**Marrow**	**1.5**	0.51 ± 0.27	0.71 ± 0.05	0.59 ± 0.12
	**24**	0.83 ± 0.17	0.81 ± 0.10	0.71 ± 0.07
	**48**	2.35 ± 0.55	1.27 ± 0.17	1.40 ± 0.22
	**120**	2.29 ± 0.61	1.72 ± 0.36	1.80 ± 0.25
**Liver**	**1.5**	4.09 ± 2.00	4.62 ± 0.62	4.65 ± 0.21
	**24**	3.21 ± 0.73	3.01 ± 0.30	3.2 ± 0.22
	**48**	1.38 ± 0.57	2.70 ± 0.12	2.74 ± 0.24

	**120**	2.05 ± 0.43	2.53 ± 0.31	2.52 ± 0.26
**Kidneys**	**1.5**	3.86 ± 1.71	6.46 ± 0.33	7.19 ± 0.86
	**24**	2.64 ± 0.24	4.84 ± 0.49	7.48 ± 3.30
	**48**	3.19 ± 0.80	5.20 ± 0.49	9.44 ± 1.19
	**120**	1.59 ± 0.14	4.46 ± 0.27	7.85 ± 0.63

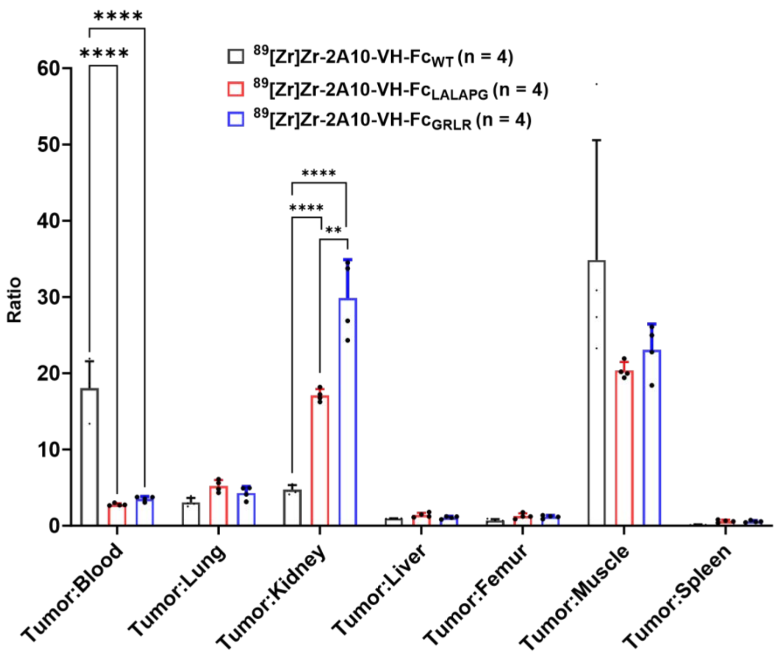

Figure S9: Bar plot demonstrating tumor-to-background ratios 5-days p.i. in HCT116 xenograft bearing mice (Female, 15 – 16 weeks). **$p < 0.01$, ****$p < 0.0001$.

**Table S8: Biodistribution (%ID/g) and tumor-to-background ratios 5-days p.i. in HCT116 xenograft bearing mice (Female, 15 – 16 weeks).**			
**Organs**	**[^{89}Zr]Zr-2A10-VH-Fc_{WT} (n = 4)**	**[^{89}Zr]Zr-2A10-VH-Fc_{LALAPG} (n = 4)**	**[^{89}Zr]Zr-2A10-VH-Fc_{GRLR} (n = 4)***
Blood	0.44 ± 0.09	3.91 ± 0.08	2.63 ± 0.35
Heart	0.95 ± 0.12	2.07 ± 0.10	1.72 ± 0.21
Lung	3.07 ± 0.59	5.21 ± 0.78	4.30 ± 0.86
Kidney	4.74 ± 0.61	17.10 ± 0.82	29.87 ± 5.04
Spleen	59.31 ± 7.62	19.42 ± 6.28	17.05 ± 3.84
Liver	8.32 ± 2.11	7.98 ± 1.45	8.73 ± 1.06
Stomach	0.52 ± 0.14	0.40 ± 0.08	0.31 ± 0.12
Intestine	1.73 ± 0.33	0.74 ± 0.04	0.63 ± 0.19
Muscle	0.25 ± 0.10	0.53 ± 0.01	0.40 ± 0.03
Femur	11.58 ± 0.37	9.04 ± 2.37	8.35 ± 2.91
Tumor	7.97 ± 2.06	10.86 ± 0.68	9.33 ± 1.79
Ovaries	7.42 ± 1.13	4.31 ± 1.29	4.23 ± 0.84
**Tumor-to-background ratios**			
Tumor/Blood	18.05 ± 3.52	2.78 ± 0.16	3.53 ± 0.32
Tumor/Lung	3.07 ± 0.59	5.21 ± 0.78	4.30 ± 0.86
Tumor/Kidney	4.74 ± 0.61	17.10 ± 0.82	29.87 ± 5.04
Tumor/Liver	0.96 ± 0.02	1.40 ± 0.31	1.07 ± 0.18
Tumor/Muscle	0.25 ± 0.10	0.53 ± 0.01	0.40 ± 0.03

** Note: One ovary sample was misplaced during biodistribution studies of the GRLR group; therefore, ovaries represent (n = 3), while all other organs represent (n = 4).*

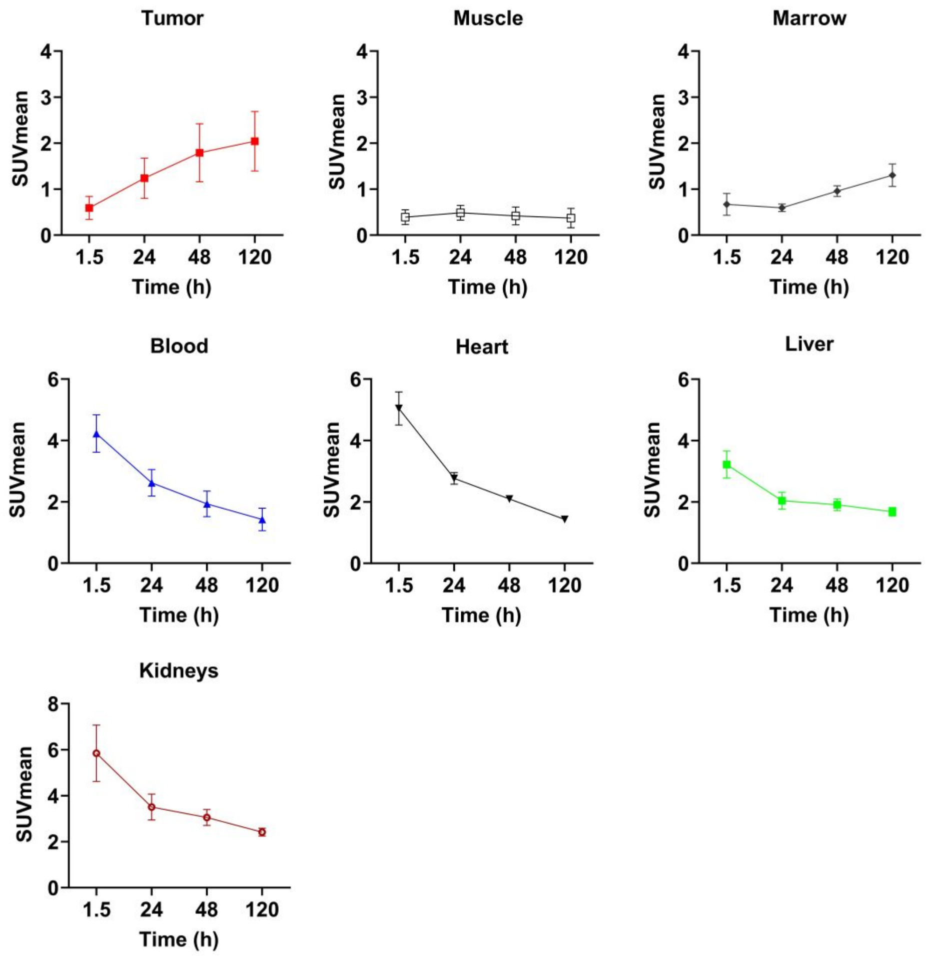

Figure S10: SUV_{mean} plot in HCT116 xenograft bearing male mice (18–19 weeks).

Table S9: SUVmean values in HCT116 xenograft bearing male mice (18–19 weeks).								
	**Time (h)**	**Tumor**	**Blood**	**Heart**	**Muscle**	**Marrow**	**Liver**	**Kidneys**
**^{89}Zr]Zr-2A10-VH-Fc$_{LALAPG}$**	**1.5**	0.59 ± 0.25	4.22 ± 0.61	5.04 ± 0.54	0.39 ± 0.16	0.67 ± 0.24	3.22 ± 0.44	5.84 ± 1.23
	**24**	1.24 ± 0.44	2.62 ± 0.43	2.76 ± 0.19	0.48 ± 0.16	0.59 ± 0.08	2.04 ± 0.28	3.50 ± 0.56
	**48**	1.79 ± 0.63	1.93 ± 0.41	2.10 ± 0.04	0.42 ± 0.19	0.96 ± 0.12	1.91 ± 0.19	3.05 ± 0.35
	**120**	2.04 ± 0.65	1.43 ± 0.37	1.43 ± 0.05	0.37 ± 0.21	1.30 ± 0.25	1.68 ± 0.14	2.42 ± 0.17

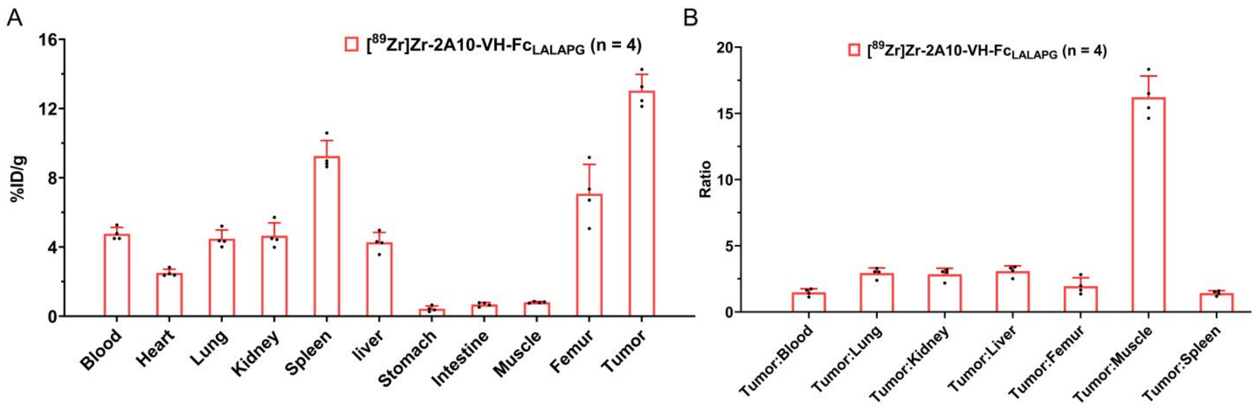

Figure S11: Bar plot demonstrating biodistribution and tumor-to-background ratios 5-days p.i. in HCT116 xenograft bearing mice (Male, 18 – 19 weeks).

**Table S10: Biodistribution (%ID/g) and tumor-to-background ratios 5-days p.i. in HCT116 xenograft bearing mice (Male, 18–19 weeks).**			
**Organs**	**[^{89}Zr]Zr-2A10-VH-Fc$_{LALAPG}$ (n = 4)**		
	**%ID/g**	**Tumor-to-background ratios**	
Blood	4.76 ± 0.37	Tumor/Blood	1.48 ± 0.27
Heart	2.50 ± 0.22	Tumor/Lung	2.94 ± 0.38
Lung	4.48 ± 0.51	Tumor/Kidney	2.85 ± 0.45
Kidney	4.65 ± 0.74	Tumor/Liver	3.09 ± 0.40
Spleen	9.25 ± 0.90	Tumor/Muscle	16.22 ± 1.60
Liver	4.27 ± 0.57		
Stomach	0.43 ± 0.16		
Intestine	0.68 ± 0.12		
Muscle	0.81 ± 0.05		
Femur	7.07 ± 1.70		
Tumor	13.03 ± 0.95		

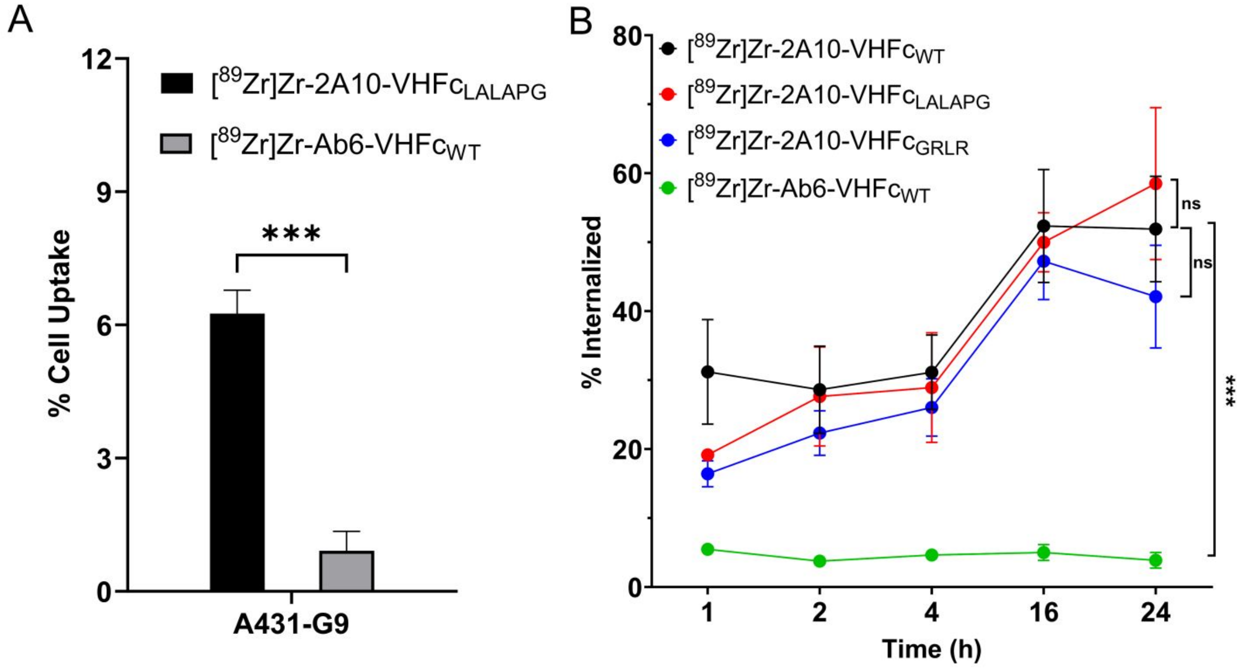

Figure S12. Cellular uptake and internalization of [89Zr]Zr-labeled VH-Fc fusion proteins in A431-G9 cells. (A) Uptake studies comparing [89Zr]Zr-2A10-VH-Fc_{LALAPG} to the non-targeting vector control [89Zr]Zr-Ab6-VH-Fc_{WT}. The Fc-mutated construct demonstrated significantly higher uptake compared to vector control (two-tailed unpaired t-test $p < 0.0002$), consistent with effective MSLN targeting. (B) Internalization kinetics over 24 h showing time-dependent tracer internalization. At 1 h, [89Zr]Zr-2A10-VH-Fc_{WT} exhibited the highest early internalization (32.55 ± 16.5%), whereas $VHFc_{LALAPG}$ and $VHFc_{GRLR}$ showed lower initial internalization (19.17 ± 0.6% and 16.42 ± 1.9%, respectively). By 24 h, internalization markedly increased for all constructs, with $VHFc_{LALAPG}$ reaching 51.43 ± 16.1%, comparable to $VHFc_{WT}$ (51.50 ± 8.4%) and higher than $VHFc_{GRLR}$ (45.39 ± 6.2%). The non-targeting control [89Zr]Zr-Ab6-$VHFc_{WT}$ remained consistently low (≤5.5 ± 0.4%) across all time points, confirming the specificity of target-mediated uptake. These results demonstrate effective cellular binding and internalization of the Fc-engineered VH-Fc proteins, supporting their potential for targeted imaging or therapeutic applications. Data represent mean ± SD, n = 3 per group; ns = not significant, ***$p < 0.001$.

**Table S11: Summary of subcutaneous xenograft models used for *in vivo* and *ex vivo* studies.**						
**Sr. No.**	**Cell type**	**Passage**	**Injected cells**	**Sex**	**Mice Age (Weeks)**	**Tested Mutants (No. of mice)**
1	HCT116	14	1×10^6	F	18 – 19	WT (n = 4), GRLR (n = 4), LALAPG (n = 3)
2	HCT116	12	1×10^6	F	15 – 16	WT (n = 4), GRLR (n = 4), LALAPG (n = 4)
3	HCT116	10	1×10^6	M	18 – 19	LALAPG (n = 4)
4	A431-G9	8	1×10^6	M	18 – 19	LALAPG (n = 5)
5	A431-G9	21	1×10^6	M	10 – 11	LALAPG (n = 4)
6	A431-G9	21	1×10^6	F	10 – 11	LALAPG (n = 4)
7	A431-H9	8	1×10^6	M	18 – 19	LALAPG (n = 4)
8	A431-H9	18	1×10^6	M	10 – 11	LALAPG (n = 4)
9	A431-H9	18	1×10^6	F	10 – 11	LALAPG (n = 4)
10	AsPC-1	11	1×10^6	M	18 – 19	LALAPG (n = 5)

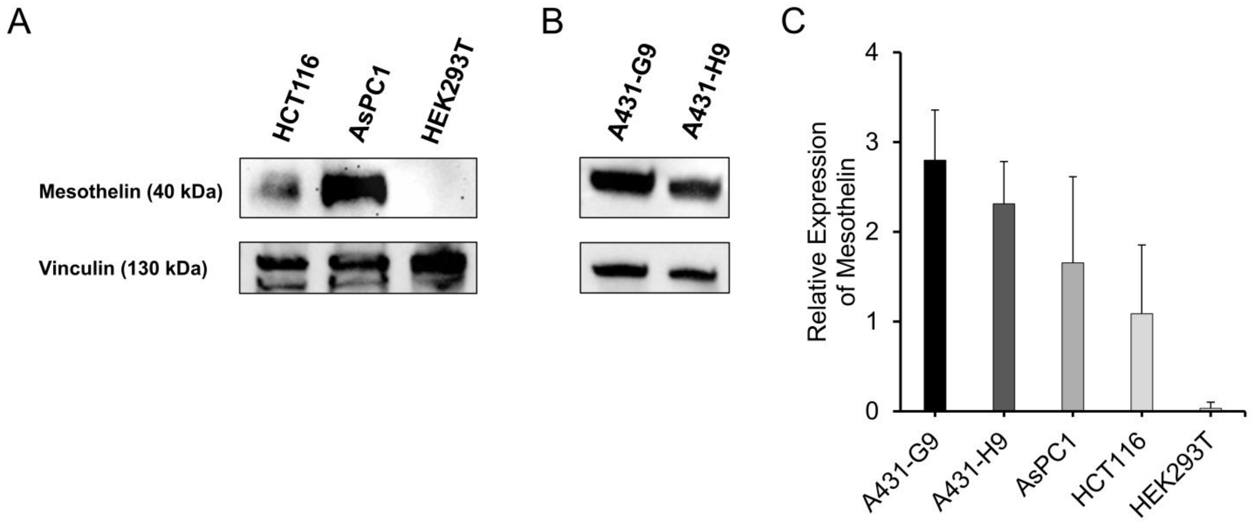

Figure S13: Representative western blot analysis showing mesothelin (MSLN) expression levels in five cancer cell lines using an MSLN-specific antibody. Vinculin was used as a loading control (A–B). Band densities were measured using Image Lab software and normalized to Vinculin expression (C). Data represents mean ± SD (n = 3).

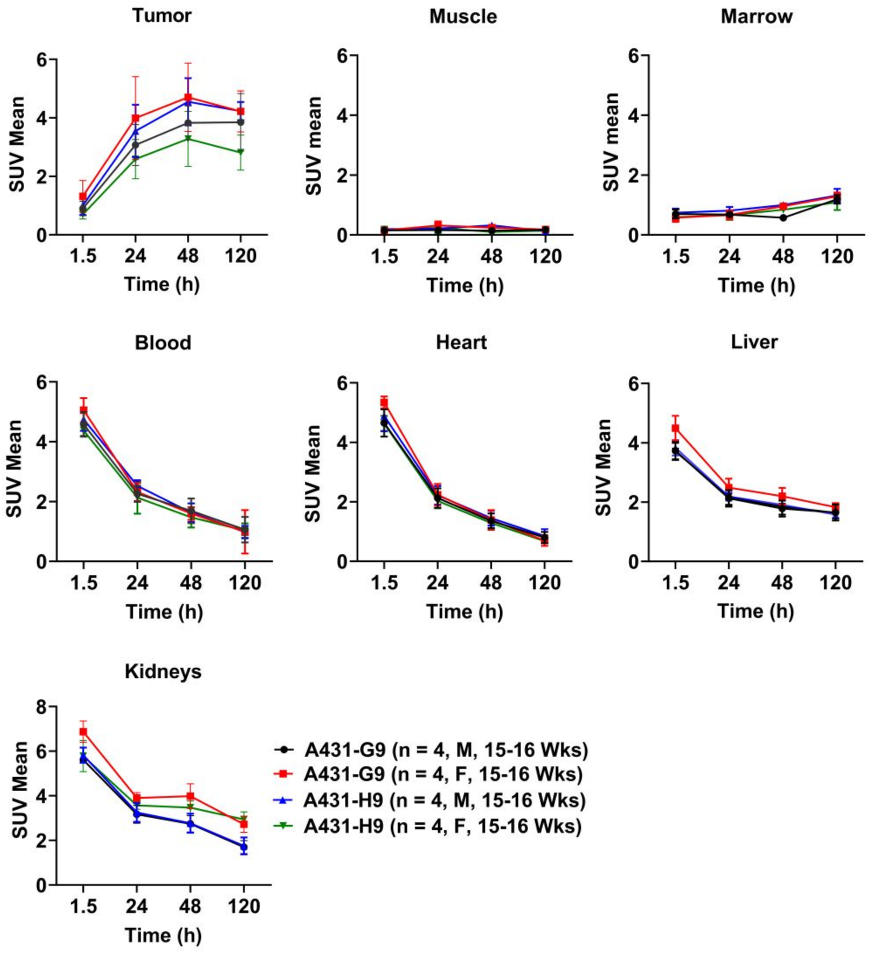

Figure S14: SUVmean values with ^{89}Zr-labeled LALAPG in A431-G9/H9 xenograft bearing male and female mice (10 – 11 weeks).

Table S12: SUVmean values with ^{89}Zr-labeled LALAPG in A431-G9/H9 xenograft bearing male and female mice (10–11 weeks).

Organs	Time (h)	A431-G9, M	A431-G9, F	A431-H9, M	A431-H9, F
**Tumor**	**1.5**	0.90 ± 0.25	1.32 ± 0.54	1.01 ± 0.33	0.70 ± 0.16
	**24**	3.07 ± 0.70	3.99 ± 1.42	3.55 ± 0.90	2.59 ± 0.67
	**48**	3.83 ± 0.58	4.70 ± 1.17	4.55 ± 0.81	3.28 ± 0.94
	**120**	3.85 ± 0.98	4.22 ± 0.70	4.23 ± 0.31	2.81 ± 0.60
**Blood**	**1.5**	4.58 ± 0.41	5.05 ± 0.41	4.75 ± 0.39	4.37 ± 0.18

	**24**	2.26 ± 0.27	2.32 ± 0.30	2.52 ± 0.19	2.13 ± 0.54
	**48**	1.69 ± 0.41	1.60 ± 0.21	1.63 ± 0.31	1.47 ± 0.34
	**120**	1.06 ± 0.43	0.99 ± 0.73	0.98 ± 0.21	1.03 ± 0.24
**Heart**	**1.5**	4.65 ± 0.46	5.34 ± 0.20	4.89 ± 0.51	4.36 ± 0.26
	**24**	2.13 ± 0.33	2.24 ± 0.36	2.22 ± 0.31	2.03 ± 0.25
	**48**	1.36 ± 0.25	1.39 ± 0.33	1.45 ± 0.25	1.29 ± 0.17
	**120**	0.80 ± 0.19	0.70 ± 0.18	0.85 ± 0.23	0.68 ± 0.07
**Muscle**	**1.5**	0.15 ± 0.06	0.15 ± 0.07	0.19 ± 0.07	0.18 ± 0.02
	**24**	0.16 ± 0.09	0.31 ± 0.14	0.21 ± 0.11	0.23 ± 0.17
	**48**	0.14 ± 0.05	0.24 ± 0.03	0.32 ± 0.11	0.10 ± 0.00
	**120**	0.17 ± 0.10	0.18 ± 0.05	0.13 ± 0.05	0.14 ± 0.07
**Marrow**	**1.5**	0.70 ± 0.15	0.57 ± 0.14	0.74 ± 0.14	1.49 ± 1.74
	**24**	0.68 ± 0.09	0.67 ± 0.15	0.81 ± 0.12	1.12 ± 0.86
	**48**	0.57 ± 0.03	0.96 ± 0.12	1.00 ± 0.02	1.14 ± 0.57
	**120**	1.19 ± 0.15	1.29 ± 0.13	1.32 ± 0.22	1.24 ± 0.42
**Liver**	**1.5**	3.73 ± 0.29	4.49 ± 0.42	3.83 ± 0.26	3.71 ± 0.29
	**24**	2.13 ± 0.28	2.49 ± 0.30	2.19 ± 0.14	2.18 ± 0.27
	**48**	1.78 ± 0.27	2.20 ± 0.28	1.91 ± 0.14	1.85 ± 0.27
	**120**	1.65 ± 0.27	1.83 ± 0.14	1.58 ± 0.12	1.64 ± 0.19
**Kidneys**	**1.5**	5.61 ± 0.14	6.87 ± 0.48	5.82 ± 0.34	5.78 ± 0.70
	**24**	3.17 ± 0.40	3.90 ± 0.24	3.25 ± 0.42	3.57 ± 0.18
	**48**	2.74 ± 0.37	3.98 ± 0.56	2.77 ± 0.43	3.47 ± 0.30
	**120**	1.70 ± 0.29	2.72 ± 0.36	1.75 ± 0.38	2.94 ± 0.34

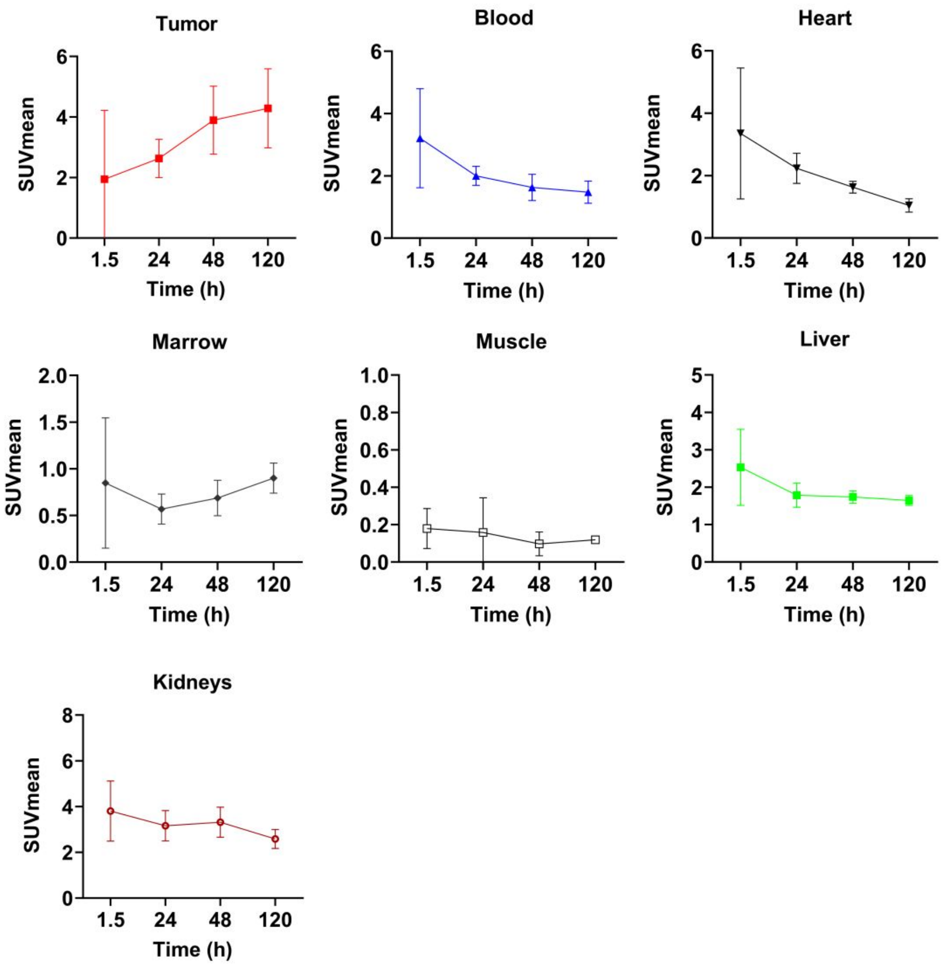

Figure S15: SUV$_{mean}$ values with ^{89}Zr-labeled LALAPG in A431-G9 xenograft bearing male mice (18–19 weeks).

**Table S13: SUVmean values with ^{89}Zr-labeled LALAPG in A431-G9 xenograft bearing male mice (18–19 weeks).**								
	**Time (h)**	**Tumor**	**Blood**	**Heart**	**Muscle**	**Marrow**	**Liver**	**Kidneys**
**^{89}Zr]Zr-2A10-VH-Fc$_{LALAPG}$**	**1.5**	1.94 ± 2.28	3.21 ± 1.59	3.35 ± 2.10	0.18 ± 0.11	0.85 ± 0.70	2.53 ± 1.02	3.80 ± 1.32
	**24**	2.63 ± 0.63	2.00 ± 0.30	2.23 ± 0.48	0.16 ± 0.19	0.57 ± 0.16	1.78 ± 0.32	3.17 ± 0.66
	**48**	3.89 ± 1.12	1.63 ± 0.42	1.63 ± 0.19	0.10 ± 0.06	0.69 ± 0.19	1.74 ± 0.16	3.32 ± 0.66
	**120**	4.28 ± 1.31	1.48 ± 0.36	1.04 ± 0.22	0.12 ± 0.02	0.90 ± 0.16	1.64 ± 0.13	2.58 ± 0.41

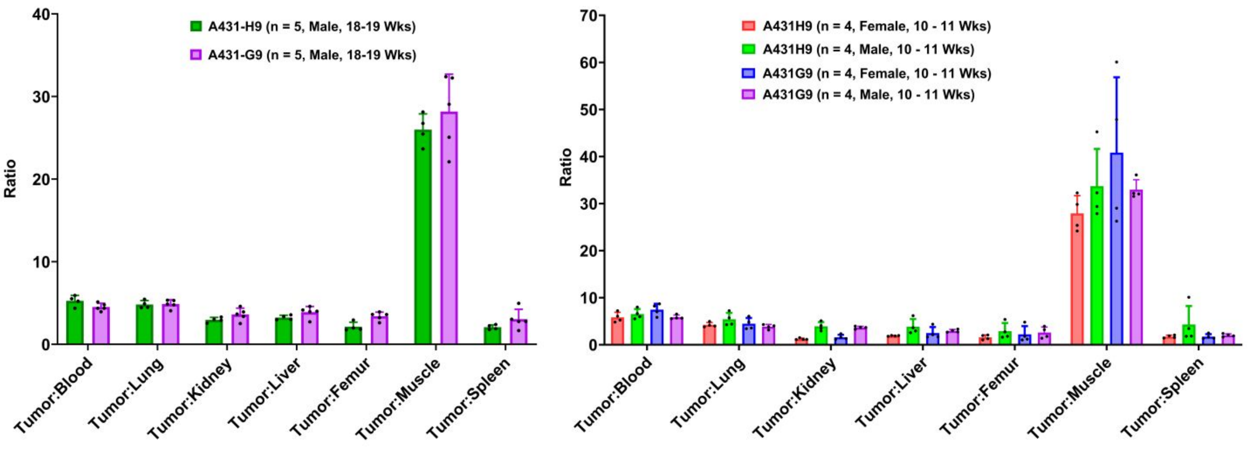

Figure S16: Tumor-to-background ratios with ^{89}Zr-labeled LALAPG in A431-G9/H9 xenograft bearing male and female mice at different age groups.

Table S14: Biodistribution (%ID/g) and tumor to background ratios 5-days p.i.						
[^{89}Zr]Zr-2A10-VH-Fc$_{LALAPG}$ (n = 4/5, %ID/g)						
	18–19 weeks		10–11 weeks			
**Organs**	**A431-H9, M**	**A431-G9, M**	**A431-H9, F**	**A431-H9, M**	**A431-G9, F**	**A431-G9, M**
Blood	2.21 ± 0.75	3.67 ± 0.95	2.04 ± 0.39	2.90 ± 0.98	2.22 ± 1.07	2.77 ± 0.84
Heart	1.49 ± 0.32	1.98 ± 0.39	1.72 ± 0.44	1.68 ± 0.55	1.60 ± 0.46	1.72 ± 0.36
Lung	2.36 ± 0.56	3.36 ± 0.66	2.79 ± 0.47	3.48 ± 0.89	3.62 ± 1.23	4.16 ± 0.79
Kidney	3.90 ± 1.15	4.54 ± 0.47	9.63 ± 1.57	4.81 ± 1.29	10.14 ± 2.79	4.37 ± 1.03
Spleen	5.54 ± 1.49	5.80 ± 1.55	6.93 ± 1.77	5.79 ± 2.14	9.53 ± 3.21	7.97 ± 1.70
Liver	3.48 ± 0.53	4.22 ± 0.65	6.10 ± 0.44	5.00 ± 0.43	6.71 ± 0.78	5.38 ± 0.93
Stomach	0.36 ± 0.11	0.39 ± 0.11	0.57 ± 0.06	0.53 ± 0.04	0.48 ± 0.18	0.47 ± 0.13
Intestine	0.55 ± 0.08	0.59 ± 0.12	0.80 ± 0.12	0.72 ± 0.10	0.77 ± 0.15	0.74 ± 0.08
Muscle	0.44 ± 0.09	0.58 ± 0.09	0.42 ± 0.06	0.56 ± 0.15	0.40 ± 0.10	0.48 ± 0.10
Femur	5.51 ± 1.62	4.93 ± 1.43	8.05 ± 3.23	7.07 ± 1.03	8.76 ± 3.16	7.50 ± 4.07
Tumor	11.29 ± 2.35	16.51 ± 4.39	11.67 ± 1.09	19.57 ± 10.07	17.20 ± 10.83	16.00 ± 3.98
Ovaries	NA	NA	3.04 ± 1.16	NA	3.89 ± 1.59	NA
Gonads	NA	NA	NA	1.20 ± 0.12	NA	1.24 ± 0.20
**Tumor-to-background ratios**						
Tumor/Blood	5.26 ± 0.66	4.52 ± 0.45	5.84 ± 1.60	6.53 ± 1.08	7.45 ± 1.29	5.86 ± 0.45
Tumor/Lung	4.82 ± 0.47	4.87 ± 0.52	4.23 ± 0.51	5.41 ± 1.38	4.47 ± 1.23	3.83 ± 0.49
Tumor/Kidney	2.95 ± 0.31	3.61 ± 0.76	1.23 ± 0.17	3.91 ± 0.91	1.60 ± 0.53	3.66 ± 0.27
Tumor/Liver	3.23 ± 0.28	3.88 ± 0.70	1.91 ± 0.09	3.83 ± 1.63	2.47 ± 1.28	2.95 ± 0.31
Tumor/Muscle	25.99 ± 1.90	28.17 ± 4.53	27.91 ± 3.78	33.69 ± 7.92	40.81 ± 16.04	32.96 ± 2.10

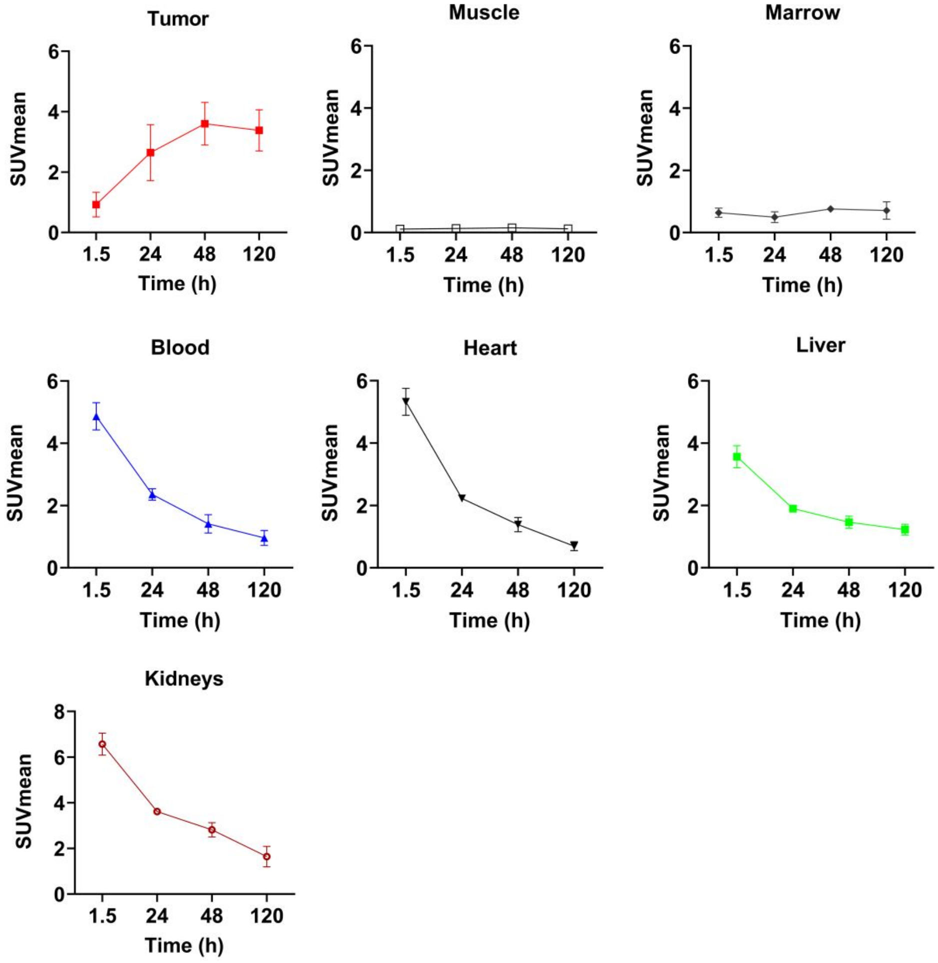

Figure S17: SUV_{mean} values with ^{89}Zr-labeled LALAPG in A431-H9 xenograft bearing male mice (18–19 weeks).

Table S15: SUVmean values with ^{89}Zr-labeled LALAPG in A431-H9 xenograft bearing male mice (18-19 weeks).								
	**Time (h)**	**Tumor**	**Blood**	**Heart**	**Muscle**	**Marrow**	**Liver**	**Kidneys**
**^{89}Zr]Zr-2A10-VH-Fc$_{LALAPG}$**	**1.5**	0.92 ± 0.40	4.86 ± 0.44	5.32 ± 0.43	0.10 ± 0.10	0.64 ± 0.15	3.56 ± 0.36	6.57 ± 0.48
	**24**	2.65 ± 0.93	2.35 ± 0.18	2.22 ± 0.08	0.13 ± 0.09	0.50 ± 0.18	1.90 ± 0.11	3.61 ± 0.10
	**48**	3.59 ± 0.71	1.41 ± 0.30	1.38 ± 0.23	0.15 ± 0.07	0.76 ± 0.08	1.46 ± 0.19	2.82 ± 0.32
	**120**	3.38 ± 0.68	0.96 ± 0.24	0.69 ± 0.14	0.12 ± 0.03	0.71 ± 0.28	1.22 ± 0.17	1.64 ± 0.45

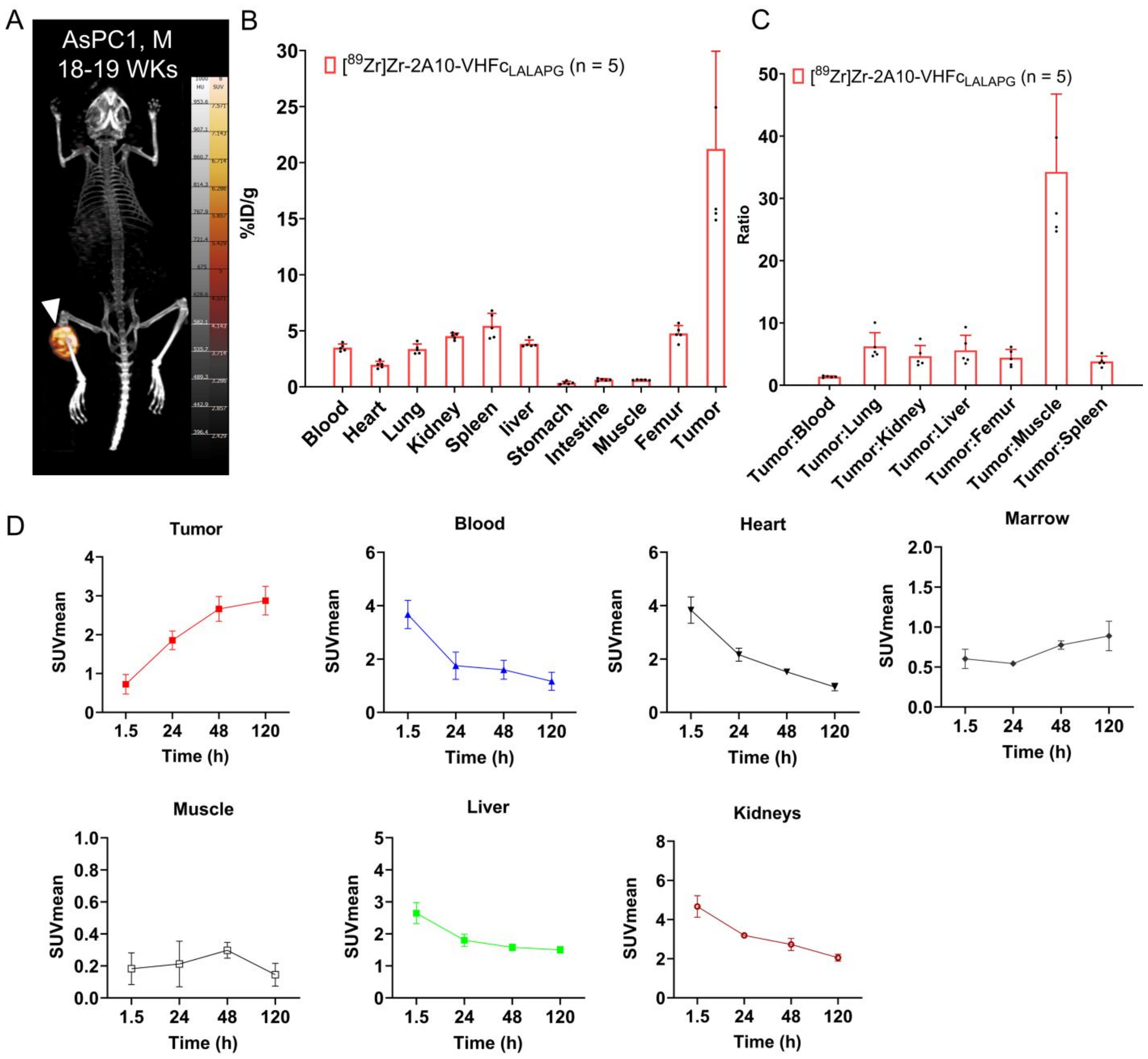

Figure S18: Representative PET image, SUVmean analysis, biodistribution, and tumor-to-background ratios in AsPC1 xenograft bearing mice (Male, 18–19 weeks) at 120 h post injection.

Table S16: SUV$_{mean}$ values in AsPC1 xenograft bearing male mice (18–19 weeks).								
	**Time (h)**	**Tumor**	**Blood**	**Heart**	**Muscle**	**Marrow**	**Liver**	**Kidneys**
**[^{89}Zr]Zr-2A10-VH-Fc$_{LALAPG}$**	**1.5**	0.72 ± 0.25	3.67 ± 0.53	3.84 ± 0.50	0.18 ± 0.10	0.60 ± 0.12	2.64 ± 0.33	4.67 ± 0.55
	**24**	1.85 ± 0.24	1.75 ± 0.52	2.16 ± 0.24	0.21 ± 0.14	0.54 ± 0.02	1.80 ± 0.19	3.18 ± 0.17
	**48**	2.66 ± 0.32	1.60 ± 0.36	1.52 ± 0.05	0.30 ± 0.05	0.77 ± 0.06	1.58 ± 0.10	2.73 ± 0.30
	**120**	2.88 ± 0.37	1.16 ± 0.34	0.95 ± 0.15	0.15 ± 0.07	0.89 ± 0.18	1.50 ± 0.10	2.05 ± 0.18

Table S17: Biodistribution (%ID/g) and tumor-to-background ratios 5-days p.i. in AsPC1 xenograft bearing mice (Male, 18 – 19 weeks).			
**Organs**	**[^{89}Zr]Zr-2A10-VH-Fc$_{LALAPG}$ (n = 5)**		
	**%ID/g**	**Tumor-to-background ratios**	
Blood	3.49 ± 0.33	Tumor/Blood	1.36 ± 0.14
Heart	1.96 ± 0.31	Tumor/Lung	6.23 ± 2.20
Lung	3.37 ± 0.45	Tumor/Kidney	4.65 ± 1.73
Kidney	4.52 ± 0.28	Tumor/Liver	5.60 ± 2.42
Spleen	5.43 ± 1.12	Tumor/Muscle	34.25 ± 12.51
Liver	3.83 ± 0.33		
Stomach	0.35 ± 0.13		
Intestine	0.63 ± 0.10		
Muscle	0.61 ± 0.03		
Femur	4.76 ± 0.70		
Tumor	21.22 ± 8.73		

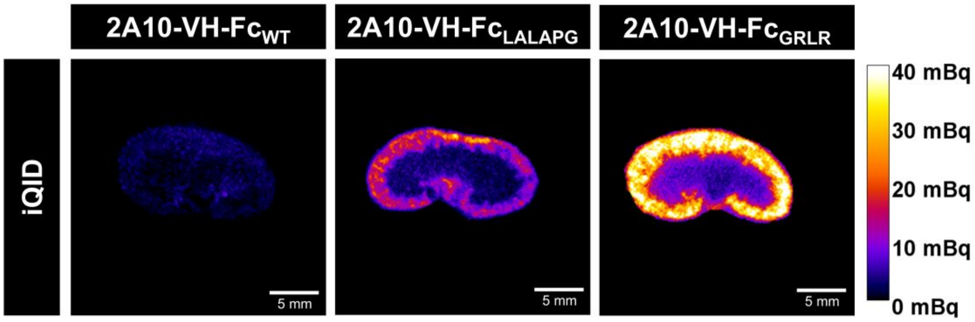

Figure S19: iQID imaging of kidneys at 24 h post injection in HCT116 xenograft bearing mice (Female, 18–19 weeks) at 24 h post injection.

REFERENCES

(1) Chu, X.; Baek, D. S.; Li, W.; Shyp, T.; Mooney, B.; Hines, M. G.; Morin, G. B.; Sorensen, P. H.; Dimitrov, D. S. Human antibodies targeting ENPP1 as candidate therapeutics for cancers. Front Immunol 2023, 14, 1070492.

(2) Chen, C.; Saville, J. W.; Marti, M. M.; Schafer, A.; Cheng, M. H.; Mannar, D.; Zhu, X.; Berezuk, A. M.; Banerjee, A.; Sobolewski, M. D.; et al. Potent and broad neutralization of SARS-CoV-2 variants of concern (VOCs) including omicron sub-lineages BA.1 and BA.2 by biparatopic human VH domains. *iScience* **2022**, *25* (8), 104798. DOI: 10.1016/j.isci.2022.104798.

(3) Sun, Z.; Jaswal, A. P.; Chu, X.; Rajkumar, H.; Cortez, A. G.; Edinger, R.; Rose, M.; Josefsson, A.; Bhise, A.; Huang, Z.; et al. Assessment of Novel Mesothelin-Specific Human Antibody Domain VH-Fc Fusion Proteins-Based PET Agents. *ACS Omega* **2023**, *8* (46), 43586-43595. DOI: 10.1021/acsomega.3c04492.
